# A serial founder effect model of phonemic diversity based on phonemic loss in low-density populations

**DOI:** 10.1371/journal.pone.0198346

**Published:** 2018-06-01

**Authors:** Joaquim Pérez-Losada, Joaquim Fort

**Affiliations:** 1 Complex Systems Laboratory, University of Girona, Maria Aurèlia Capmany 61, Girona, Catalonia, Spain; 2 Catalan Institute for Research and Advanced Studies (ICREA), Lluís Companys 23, Barcelona, Catalonia, Spain; Indiana University Bloomington, UNITED STATES

## Abstract

It has been observed that the number of phonemes in languages in use today tends to decrease with increasing distance from Africa. A previous formal model has recently reproduced the observed cline, but under two strong assumptions. Here we tackle the question of whether an alternative explanation for the worldwide phonemic cline is possible, by using alternative assumptions. The answer is affirmative. We show this by formalizing a proposal, following Atkinson, that this pattern may be due to a repeated bottleneck effect and phonemic loss. In our simulations, low-density populations lose phonemes during the Out-of-Africa dispersal of modern humans. Our results reproduce the observed global cline for the number of phonemes. In addition, we also detect a cline of phonemic diversity and reproduce it using our simulation model. We suggest how future work could determine whether the previous model or the new one (or even a combination of them) is valid. Simulations also show that the clines can still be present even 300 kyr after the Out-of-Africa dispersal, which is contrary to some previous claims which were not supported by numerical simulations.

## 1. Introduction

How human language began is one of the greatest questions posed to the humankind, to which an answer has yet to be found [1–3]. Some authors have argued that principles and processes of genetic evolution (such as migration, population divergence, and drift) are, with appropriate modifications, valid for explaining the evolution and the origin of languages [4,5]. However, genetic and linguistic evolution do not inevitably concur [6]. On the other hand, genomic and archaeological studies have shed light on the spatiotemporal dynamics of modern humans, their origin and patterns of dispersal(s) [7,8]. In contrast, the study of the origin of language(s), in the field of language evolution, is controversial for the reason that we lack any direct data about the language(s) spoken in such remote times [2,9,10].

At present, most of the languages with the largest phonemic inventories are in Africa. By contrast, South American and Oceanian languages have the smallest inventories [11,12]. Atkinson [11] attempted to connect this observation to the fact that, according to archaeologic evidence [8], modern humans left Africa and eventually arrived to South America and Oceania. Analogously to the serial founder effect (SFE) proposed to explain the observed decrease in *genetic* diversity with distance from Africa [13,14], Atkinson [11] proposed that the same mechanism of SFE could also explain the formation of a global *phonemic* cline during the Out-of-Africa human dispersal about 70 kyr ago [7,8]. According to Atkinson [11], the proposed SFE mechanism would work as follows: small populations of humans carrying their language(s) moved away from their origin in Africa and founded colonies. The number of phonemes of the languages spoken by these small colonies decreased. This may have been due to copying errors [15], Darwinian competition [16], reduced contrastive possibilities [17], and/or to other processes (see Supp. Info. to Ref. [11]). The repetition of this decrease furthers the reduction in the number of phonemes in the languages spoken by the colonizer populations during the expansion. Thus, although the correlation of the number of phonemes with distance from Africa is clearly weaker (*r* = -0.313, *p* < 0.001) [18] than the relationship between genetic distance and geographic distance (*r* = -0.885, *p* < 0.0001 from Fig 1B in Ref. [13]), the Out-of-Africa expansion might perhaps be the process that produced both clines [19]. Atkinson located the origin of human language in Africa, at the place yielding the best linear fit to a measure of phonemic inventory size versus distance from the origin. If his suggestion is correct, it follows that, in spite of tens of thousands of years of linguistic evolution, there is still a weak signal carried on the phonemes of languages spoken today that may help us understand the spatiotemporal dynamics of human language.

The proposal by Atkinson that the worldwide phonemic cline could be a consequence of a SFE, was also motivated by the fact that Hay and Bauer [20] had previously discovered a worldwide positive correlation between the speaker population size and the number of phonemes in human languages. Albeit Hay and Bauer [20] tested it statistically, they recognized that they could not find a convincing explanation for it. Atkinson checked a positive correlation between population size and phonemic inventory size (S1 Fig in Ref. [11]). Donohue and Nichols [21], using a different phonemic dataset, again found a correlation between population size and phonemic inventory size, by aggregating languages into continent-sized areas, but suggested that it might be due to different political and economic histories of continents over the last two millennia. However, they did not use any quantitative simulation to support their view. Therefore, there is still an ongoing debate on whether the number of phonemes is positively correlated with population size. At the heart of the debate lies the question whether there is a link between population size and cultural complexity [22–24]. In order to avoid confusion, we mention that all of these studies refer to a possible relationship between cultural complexity and population size. In contrast, our simulations (both in Ref. [18] and the present paper) do not make use of population size but of population density as the variable that might induce variation in the number of phonemes (because otherwise SFE models do not yield a cline of decreasing phoneme number with increasing distance, see Supp. Info. Sec S2d in Ref. [18]. On the other hand, Maddieson et al. [25] noted that Atkinson did not use data on either the number of phonemes or their diversity. Indeed, Atkinson used the same WALS phonemic database [26], which only classifies languages into, e.g., a small, medium and large number of vowels. Instead, Maddieson et al. [25] used raw phoneme counts. They found once again a significant positive correlation between phonemic inventory size and geographical distance, which supports the cline detected by Atkinson. Cysouw et al. [27] replicated the same statistical method as Atkinson (using the UPSID [28] and reweighted WALS phonetic databases) and found a positive correlation, but only for populations larger than the ones presumably found at the time of the Out-of-Africa dispersal. The same criticism was made by Sproat [29]. Atkinson replied that this may be due to historical distortions and reducing the databases to few data points, and that the correlation over the complete range holds [30,31]. Finally, Trudgill [17] argued that migration does not lead *per se* to a reduction in the inventory size, and that a combination of social and linguistic factors could perhaps explain the observed reduction in phonemes during the spread of Austronesian languages [32].

Pericliev [33], Sproat [29] and Bowern [34] raised concerns about the application of a SFE to explain the global phonemic cline, because of a lack of direct analogy between phonemes and genes. Indeed, the same language is shared by all individuals of a population, but they do not have the same genes. However, this difference is taken into account in our simulations (Ref. [18] and the present paper) because we deal with tribes, and all individuals in the same tribe speak the same language. On the other hand, Atkison [30] argued that the reduction of phonemes after a founder effect is predicted by theoretical models of cultural transmission [15–17]. By contrast, the loss of genetic markers in low-density populations is purely a drift effect. Therefore, the processes of reduction in phonemes are different from those of genes, and this implies that there is no direct analogy between founder effects in genetics and phonemics.

The location by Atkinson of the origin of human languages in Africa was challenged by Cysouw et al. [27], whose analysis located their origin most probably outside Africa. But, Atkinson [31] replied that, when accounting for population size and language affiliation, Cysouw et al. [27] also found support for an African origin. On the other hand, Wang et al. [35] found the strongest relationship for two origins, one in Europe and one in central Asia. However, Atkinson [31] noted that the analysis by Wang et al. [35] depended heavily on four closely related outlier languages. When removed from the dataset, the most probable location of the point of origin continues to be located in Africa.

The acoustic adaptation hypothesis [36] assumes that animal and human communication systems are adapted to environment and climate. Maddieson et al. [25] suggested that phoneme articulation in human languages operates similarly, as a possible explanation for the global phonemic correlation. Indeed, the observed pattern of the number of phonemes is affected by environmental and social factors in different ways [37]. However, Coupé et al. [38] used the same dataset as Atkinson [11] and correlated the phonemic diversity with environmental and social variables. After all the environmental factors were included in the regression analysis, Coupé et al. [38] found that the distance from Atkinson’s most likely origin was still a strongly significant factor, thus rejecting the hypothesis that a coincidental distribution of local factors could explain the global phonemic gradient.

Finally, a more fundamental criticism of the Atkinson hypothesis is the stability of the phonemic signal. Several criticisms assume that high rates of phonemic change would have erased any signal due to a SFE during the Out-of-Africa range expansion [27,29,34,39–42]. Atkinson responded that the phonemic inventory is stable at the language-family level [30,31]. Atkinson also defended that, as in the case in population genetics [43] horizontal transfer due to borrowing of phonemes between adjacent languages (after the dispersal) has not disrupted the original SFE signal but, on the contrary, has helped to preserve it. It is also interesting that Atkinson [31] argued there is no motivated reason to expect that other typological features of language show a founder effect (the absence of such additional clines had been suggested by Cysouw et al. [27] as a reason to reject the SFE hypothesis).

The key issues that we will address in the present paper (using numerical simulations) are the following. First, can we generate the observed cline for the number of phonemes as a function of distance from Africa using a model based on the proposal by Atkinson that low-density populations lose phonemes [11]? Second, is there a cline of phonemic diversity (and not only of the number of phonemes) in present languages? Third, if the answer is affirmative, can the same model generate such a phonemic diversity cline? Fourth, do both clines persist after long enough times to be observed today, according to the same model? We shall find that the answers to all of these questions are affirmative.

Recently we have shown [18] that the hypothesis that a phonemic SFE could have caused the observed spatial phonemic cline can be tested quantitatively with linguistic simulations based on formal models, following a similar approach found in genetic simulations [13,14] but taking into account the remarkable differences between phonemic and genetic dynamics [18]. A crucial feature of numerical simulations is that they replace explanations based on natural language with formal models that can explore the conditions and mechanisms that could have generated (or not) the observed non-uniform global phonemic distribution.

In our recent paper [18], we reported numerical simulations for a phonemic SFE model by applying the idea, proposed by Perrault and Mathew [44], that some populations increase the number of phonemes used over time. We introduced four models, explored different rates of phonemic increase, and tested several hypotheses on the relationship between the rate of accumulation (or increase) of phonemes and speaker density. A model assuming that only languages with high speaker densities increase their number of phonemes (without assuming any specific cultural transmission mechanism) was enough to explain the observed global phonemic cline. These models in Ref. [18] are very simple and overcome several criticisms raised against the proposal by Atkinson (mainly based on differences between phonemes and genes, see above). However, the model that yields a cline consistent with the observed one [18] relies on two strong and untested assumptions: (i) that languages at the onset of the Out-of-Africa dispersal had low phonemic inventories (as also assumed by Perrault and Mathew [44]); and (ii) the existence of a phonemic accumulation rate, as estimated by Perrault and Mathew [44] from phonemic and archaeological data. In this paper, we ask the following question: is it possible to find an alternative model to that phonemic cline by making assumptions different to these used in Ref. [18] and summarized above? We shall show that the answer is affirmative, and this will clarify the possible mechanisms that might have generated phonemic the observed phonemic cline.

A close examination of the two assumptions in Ref. [18], summarized above, casts some light on how a different SFE model can be constructed. Firstly, the number of phonemes of the language(s) spoken at the onset of the range expansion is unknown. The rationale implicit behind assumption (i) above is that languages at the onset of the range expansion might be simple, with few phonemes. However, it has been argued that present African languages (especially click languages) could display features of the ancestral “mother tongue” [1,45]. In this framework, Fleming [46] outlined a possible explanation of the observed global phonemic cline. He proposed that languages at the origin of the range expansion had a much larger phonemic inventory than commonly assumed. In his view, a large phonemic inventory reflects the archaic signal of a protolanguage, preserved through sustained linguistic contact between language groups employing a large phonemic inventory. Fleming also suggested that once the horizontal transmission between languages is lost, as could be the case of a SFE, the phonemic inventory would go through a process of reduction. On this account, Fleming places the location of the origin of human language at the click languages area in southern Africa, which has languages with the largest phonemic inventories in the world [47]. However, Fleming did not perform any simulation to support his views quantitatively. In this paper we will explore a simple simulation model consistent with large initial phonemic inventories before the Out-of-Africa dispersal. Remarkably, we do not need any of the specific assumptions made by Fleming [46] other than a reduction in phonemic diversity in populations with low speaker densities (as already suggested by Atkinson [11]).

A second strong assumption in our previous simulations [18] was a natural rate of increase of phonemic inventory size, but the existence of such an accumulation rate is uncertain [48]. Therefore, it is reasonable to ask whether the observed cline can be reproduced by a model different from that in Ref. [18], i.e., without small initial inventories and without a phonemic accumulation rate. In fact, it has been argued [29] that a phonemic cline can arise from a SFE either because high-density populations gain phonemes (as in Ref. [18]) or because low-density populations lose them (as in the model that we will develop in the present paper). This second case, i.e., the loss of phonemes in low-density populations, corresponds to the original hypothesis by Atkinson [11,30], and is the alternative possibility that we explore in the present paper. Although there is no direct proof that low-density populations loose phonemic diversity, proposals to explain many archaeological and social phenomena consider that populations with low densities tend to have small cultural diversities. This notion has been used to understand the spatial distribution of mode 1 and mode 2 assemblages during the lower Pleistocene [49], the transition to modern human behavior [50], the appearance of social stratification and regional institutions [51], etc. Empirical data for non-industrial societies show that cultural diversity increases with population size [52]. Laboratory experiments have also shown that smaller human populations have less cultural complexity [53]. Moreover smaller populations have been observed to have higher rates of word loss [54]. On the other hand, phonemic diversity has been observed to be lower if there is less contact with other languages [38]. During the Out-of-Africa dispersal, this effect is expected to be important in pioneering groups, because they are obviously much less exposed to other populations and languages [38]. In any case, the fact that small populations tend to have fewer phonemes has been established statistically [20], including analyses which control for genealogical relatedness [11] and use larger datasets [55]. Therefore, the assumption of a loss of phonemes in low-density populations has reasonable empirical grounds.

In our opinion, we can be confident in the existence of the cline reported by Atkinson and its slope, because in our previous paper (Ref. [18], S2 Software, Sec S1) we analyzed two other databases (one due to Ruhlen, already used in Ref. [56], and another one due to Hunley et al. [41]) and obtained almost the same slope. Creanza et al. [56] found that the *AIC* statistic (instead of *R*, which was used by Atkinson) reaches its maximum in Northern Europe. The result has been used to criticize that Atkinson proposed an origin in Africa [57]. However, as noted by Creanza et al. [56], their most likely origin is in a region (Northern Europe) that is equidistant from most languages with few phonemes, which are located in Oceania and South America. In contrast, if considering their origin to be in Africa, languages in Oceania are at shorter distances than languages in South America. But human dispersal in Oceania took place via sea travel, and this fact and the higher isolation of those islands (as compared to populations in continental South America) may have had significant effects on linguistic evolution that could perhaps explain the similar values for the number of phonemes in Oceania and South America (and, thus, the lower *AIC* value from Africa than from Northern Europe). Of course, we do not claim that this is the only possible explanation. However, in our opinion, the important point is that archaeological and genetic evidence clearly indicate that modern humans spread over the Earth from Africa [13,58]. For this reason, we think that it is reasonable to use the origin proposed by Atkinson. It is also worth to note that the values of the slope, *r* and *p*, of the phonemic cline discovered by Atkinson cannot be directly compared to the corresponding values of the genetic diversity cline [13,14] because they are due to totally different mechanisms. Indeed, in the present paper we will present a model in which the phonemic cline arises as a consequence of the loss of phonemes (as well as stochastic drift effects), but there is no loss of genetic markers due to any mechanisms different from stochastic drift [59]. In other words, when a sub-population separates from a larger population, all phonemes are used but some genes can be lost. In our view, such remarkable differences between phonemes and genes suggest that we should not expect the same results between them. In particular, we do not expect that the reported differences in correlations of phonemic and genetic distances [56] are enough to disregard the possibility that a SFE caused the phonemic cline discovered by Atkinson. Similarly, the recent claim that vocabulary items do not preserve a deep historical signal [60] does not imply that such a signal has been necessarily erased for phonemes, since different linguistic features (such as vocabulary, phonemes, grammar, etc.) evolve at different rates [61].

## 2. Methods

### 2.1 The observed phonemic cline

The data we use are, for each language, its number of phonemes and the distance from the African origin proposed by Atkinson (S1 Database). For the sake of clarity, we mention that Atkinson [11] used the WALS database [26] for his analysis. However, WALS does not report the list of phonemes for each language, but only distinguishes between languages with low, average and large diversity (for vowels, consonants and tonality). For the purpose of our simulations, we need the list of phonemes of each language and, for this reason, in Ref. [18] and this paper we use the Phonological Segment Inventory Database (UPSID) database [28]. From the original list of 504 languages given by Atkinson [11], in Ref. [18] we identified 366 languages also present in UPSID; of these languages, we identify 359 with the detailed lists of their phonemes. In Ref. [18] we checked that the global cline of phonemic diversity is still present in the reduced dataset (Fig 1 in Ref. [18]).

### 2.2 The simulated phonemic cline

Our model simulates the dispersal of hunter-gather tribes from a single origin. These hunter-gatherers reproduced, dispersed and died. Their descendants gradually colonized the Earth, spreading evolved forms of the original languages spoken at the onset of the dispersal. As explained in the introduction to this paper, we assume that the languages of low-density populations evolved by randomly losing phonemes. Our SFE model uses a set of parameters. Obtaining good estimates of the parameter values is a daunting task. Here, we also face the problem that there is no information on the languages spoken at the onset of the Out-of-Africa dispersal [2,9]. One way of reducing uncertainty is to obtain the values of as many parameters as possible from the ethnographic bibliography. We follow this approach, as explained in detail below.

We represent the surface of the Earth as a two-dimensional space, sliced in a grid of squared cells. The center of each cell is called a node. In our simulation there are 1,000 x 1,000 nodes. Each cell may have some tribes of hunter-gatherers. The number of tribes in each cell can change each generation, due to net reproduction and/or dispersal. Geographical landmasses such as oceans and mountain ranges are not included for simplicity (i.e., we prescribe a homogeneous space). For the distance between any two adjacent nodes, we use the characteristic dispersal distance per generation for pre-industrial populations, namely *d* = 50 km, which has been estimated previously from ethnographic observations [62]. Initially, only the central cell is populated by some tribes of hunter-gatherers (but the results would be similar if several cells were initially populated).

Along the horizontal (or vertical) direction, the maximum distance from the origin (which is located at the center of the square grid) to the edges of the simulated two-dimensional space is 500·50 = 25,000 km, which is similar to the maximum distances from Africa of the languages in the dataset used.

Population density estimations of hunter-gatherers vary widely [63]. We choose a representative value of 1.2 people/km^2^, within the range reported for populations in various continents [63]. In agreement with ethnographic observations, a tribe is defined as a highly endogamous reproductive group of about 300 people with a common language [64]. In our model, each person speaks only one language. All people of each tribe speak the same language. Several tribes, possibly located in different cells, may speak the same language. For our grid of cells (of 50km x 50km = 2,500 km^2^ each) and a population density of 1.2 people/km^2^, this implies that each cell can accommodate a number of tribes *N* less or equal to the saturation value, namely *N*_*s*_ = 10 tribes. Of course, other ethnographically realistic values for the population density and number of individuals per tribe are possible. In S1 Text, Sec G we find similar results to those reported below for other ethnographically realistic values of *N*_*s*_. The language of each tribe is encoded in a binary string of digits equal to 0 (absence of a particular phoneme) or 1 (presence of that phoneme), as explained in more detail in Sec 3.2.

A quantitative description of the dynamics of human dispersal requires using the generation time (*T*), defined as the mean parent-child age difference [65]. In our model, each time step *T* represents one generation of *T* = 32 y, as estimated for pre-industrial populations [65]. Front propagation models have been extensively applied to the study of physical and biological systems, including human dispersals [66–68]. In such systems, variations in the population number density are due to two processes, namely population growth (reproduction minus deaths) and migration (dispersal). As in previous simulations of space-time cultural diversity [18,69], the following sequence of computations is executed at each occupied node and for each time step.

(i) Dispersal. For simplicity, we used an isotropic single-distance dispersal model [62,69]. In this model, a randomly-selected fraction of tribes (called the persistence) stays at the original cell. We set the persistence to its mean value, as observed from pre-industrial ethnographic observations [62], namely, *p*_*e*_ = 0.38. We approximate the number of tribes that stay in the original cell to the nearest integer of the product of the initial number of tribes times the persistence (e.g., if the initial number is 10 tribes, then 4 of them stay). The remainder fraction, i.e., the nearest integer of the initial number of tribes times (1 − *p*_*e*_)/4, disperses randomly into each of the 4 neighboring nodes. Thus, in the same example, of the 6 tribes that change their position, 4 of them jump, one into each neighboring cell, and the other 2 into one or two of these 4 neighboring cells.
(ii) Reproduction. From archaeological data, the net reproductive rate (births minus deaths) can be estimated. Accordingly, we set the reproductive rate to an average value of 1*%*, i.e., *a* = 0.01/y [70]. From the values of *a* and *T* given above, for an exponential growth (i.e., for low population densities) we have *p*(*x*,*y*,*t* + *T*) = *e*^*aT*^*p*(*x*,*y*,*t*), with *e*^*aT*^ = 1.4 or *R*_0_ = 1.4 (where *R*_0_ = *e*^*aT*^ stands for the net fecundity, in accordance with the usual notation in the ecological literature and in our previous work [62]). This simply states that at each node, new tribes are generated by multiplying 1.4 times the initial number of tribes. The resulting number is adjusted to the nearest integer. This implementation would, however, yield an infinite growth that should be limited by the carrying capacity. For this reason, at each node, if we obtain a number of tribes above the carrying capacity (10 tribes per node, see above), the number of tribes is set equal to the carrying capacity. An alternative would be to use a logistic model, but we expect that the conclusions would not change. Another advantage of using the same approach (for steps (i)-(iii)) as in Ref. [18] is that in this way, differences in the results will be surely due to differences in the assumptions mentioned in the introduction.
(iii) Vertical transmission. In this step, information is passed from one generation to the next one without any change (i.e., each new tribe is a clone of its parent tribe), as in Refs. [69,70]. The information on the number of phonemes of a given language, stored in the sequence of “0”s or “1”s (indicating the absence or presence of each phoneme), is copied from a randomly-selected old tribe (‘parent’) onto a randomly-selected new tribe (‘child’). Note that this process does not generate new languages.
(iv) Mutation. Each tribe has one language, whose phonemes are represented by a string that can change in time. In order for simulations to be faster, for initial languages each string is a list of “1” values, followed by a list of “0” values. As mentioned in the introduction, in Ref. [18] we assumed that high-density populations increase their number of phonemes, whereas here we assume that low-density languages lose phonemes. Our model does not assume nor exclude any particular mechanisms by which languages lose phonemes. Instead, a single averaged rate of reduction of phonemes, that describes the net effect of these mechanisms (for low-density populations) on the phonemic inventory, is prescribed. This process is simply a phonemic reduction that turns a value “1” (presence of a phoneme), chosen at random in the ‘parent’ language, into a “0” (absence of that phoneme) in the ‘child’ language. Note that after the first mutation (loss of a phoneme), the language is not necessarily a list of “1”s followed by a list of “0”s, because the “1” that has changed into a “0” is not necessarily the last one (this is necessary to compute phonemic diversities, as explained in Sec 3.2 below). In this way, new languages appear at the nodes with density below saturation i.e., with *N* < 10 tribes (in S1 Text, Sec F, we find similar results for other values of this threshold i.e., *N* = 9, *N* = 8, etc.). Note that those low-density nodes are located at the front of the advancing wave. We stress that, whereas one generation is the time interval for steps (i)-(iii), the phonemic loss process takes place at a time interval greater that one generation. The phonemic loss time is an unknown parameter that is calibrated by trial and error (see the results below). Our simulation code is provided as a separate file in S1 Software.

Is this mutation step necessary? The effect of drift on small groups has been already established for neutral traits [71,72]. However, the effect of drift on populations at the propagation front is not in itself informative on the possibility that founder events alone can generate a distribution comparable to the one observed in the empirical data. In our model, loss of phonemes is mutation-based rather than fission-based. Besides fission (i.e., the dispersal step) which basically acts on the number of different languages at each node, there is also the additional effect of mutation, which reduces the number of phonemes in low-density cells. For this reason, in S1 Text, Sec H we consider the case of an infinite phonemic time loss, i.e., the total absence of an effect of mutation (phoneme loss) on the number of phonemes, and we conclude that, for a phonemic cline with distance to exist, a finite phonemic time loss is necessary (i.e., mutation is required in addition to drift).

The algorithm proceeds sequentially by computing first the dispersal, then the reproduction, and finally the vertical transmission processes. The mutation process is also applied but, as mentioned above, not to every generation. A reasonable estimation of the time span from the beginning of the Out-of-Africa dispersal up to the present day is of about 70 kyr [7] (although how and when it occurred is still debated [73]). With this uncertainty in mind, we run the model 2,280 generations x 32 y/generation = 72,960 y. We expect that other realistic dates would yield similar results (as in Ref. [18]). Once a tribe reaches the end of the grid space, it cannot go further. Two boundary conditions are thus applied, namely at the nodes located at the edges of the square grid (which exchange tribes with 3 neighbors) and the 4 nodes located at the corners (which exchange tribes only with 2 neighbors). We have checked that, for the values of *T*, *p*_*e*_ and *R*_*o*_ above, the hunter-gatherer population front arrives to Australia about 42 kyr ago [18], in agreement with archaeological data [74].

Note that the process is stochastic (see steps (i)—(iv) above), so that our simulations yield different results every run, even for the same parameter values. We performed 100 simulation runs for each parameter set (each run took 4 to 5 hours of computing time).

The initial conditions at the beginning of the simulations (i.e., at the onset of the Out-of-Africa dispersal) include the number of tribes and their phonemic inventories at the central cell of the grid. The typology and linguistic features of these archaic languages are unfortunately unknown [2,9], so we shall consider two reasonable scenarios below. All nodes but the central one are uninhabited at the start of the simulations. The central node was initially seeded with *N* = 10 tribes (see above). We consider two possible initial conditions.

(a) We firstly explore the possibility that languages at the onset of the range expansion had fewer phonemes than present-day click languages. In this possibility (a), we use languages within a range of 35 to 40 phonemes, which is the range implied by the observed present cline in the region of origin of the Out-of-Africa dispersal i.e., the intercept (and its 95% confidence-level error) of the plot of the number of phonemes versus their distances from the most likely origin of the Out-of-Africa dispersal, see Fig 1 in Ref. [18]. The central node is initially populated with 10 tribes and 5 initial languages (there are 2 initial tribes for each language), and each tribe speaks one language with a number of phonemes between 35 and 40.
(b) Secondly, we take into account what Fleming [46] proposed: that languages with a very high number of click sounds are a feature preserved from an early state of language evolution. Under this hypothesis, and in the absence of any prehistoric linguistic data, it is reasonable to assume that the number of phonemes of the original languages were similar to those of the present-day languages with the largest phoneme inventories. Present-day click languages have the largest number of phonemes [44]. Table 2 in Perrault and Mathew [44] reports a mean value of 71 phonemes for five extant click languages. Thus in the initial conditions (b), we assume that languages at the onset of the Out-of-Africa range expansion possessed around 71 phonemes. All people of each tribe speak one language with 66 to 76 phonemes. As explained above, there are 10 initial tribes and 5 initial languages (thus, there are 2 initial tribes for each language). These numbers of phonemes correspond to the proposal by Fleming that the number of phonemes of initial languages before the Out-of-Africa dispersal were similar to those of present-day click languages [46].

Additionally, to check our results, we also repeated the simulations assuming that all initial tribes spoke the same language for the two cases studied ((a) and (b) above, with 37 and 71 phonemes, respectively) and found similar results (see below).

In our SFE model, languages lose phonemes by randomly subtracting one phoneme from each language (i.e., a digit “1” is turned into “0”) only in regions (nodes) where the population is below the saturation density (i.e., only in nodes with *N*<10 tribes), at the prescribed phonemic loss time. Languages in these nodes lose phonemes, regardless of whether all those 10 languages are the same or not. For example, in a cell with 3 tribes, we can have 1, 2, or 3 different languages, and all of them lose phonemes. The results are similar for other values of the population threshold density of phonemic loss, as well as for other values of the population saturation density (see S1 Text, sections F and G, respectively).

Conceptually, our model works as follows. As explained above, phonemes are lost in recently-colonized regions (cells), the population densities of which are low. Such regions (cells) are located at the front of the wave of advance of the pioneering populations of modern humans. Thus the mechanisms of propagation of the front (dispersion and reproduction) further disseminate these pioneering populations speaking low-diversity languages. In contrast, regions near the origin (and other regions that lie behind the front) have already reached their maximum population density, therefore they are not affected by the phonemic reduction process. This bottleneck effect is repeated many times and, as we shall see, gives rise to a cline of a decreasing number of phonemes per language as a function of distance from the origin of the dispersal.

## 3. Results and discussion

### 3.1 Cline for the number of phonemes

**Fig 1:**
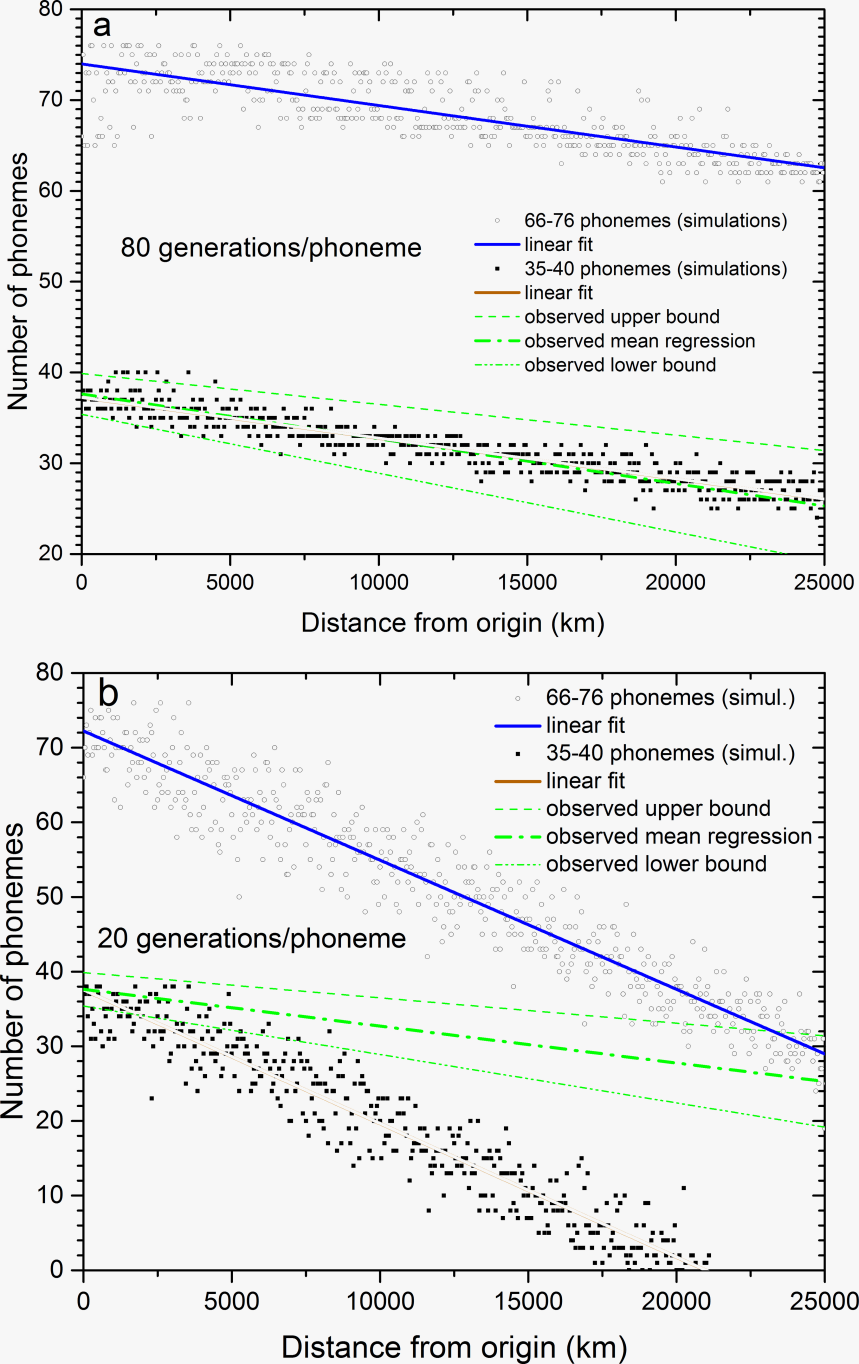
Simulated phonemic cline (i.e., number of phonemes versus distance from Atkinson’s putative origin of the Out-of-Africa dispersal). Two instances of the same model are shown in which at the onset of the Out-of-Africa dispersal, initial languages had 66–76 phonemes (open circles) and 35–40 phonemes (squares). The observed global phonemic cline [11] is also shown (green dash-dotted and dashed lines). The initial population is made of *N* = 10 tribes at the central cell speaking a mix of languages, and the saturation density and demographic threshold (below which phonemes are lost) are set at *N* = 10 tribes per cell. Net fecundity *R*_*0*_ = 1.4. Time = 2,280 generations or about 70 kyr, corresponding to the present time from the Out-of-Africa dispersal. a) Phonemic loss time equal to 80 generations/phoneme (i.e., 1 phoneme disappears every 80 generations in low-density populations). For both regressions (blue and red solid lines), the simulated slope is -(4.3–4.9)·10^−4^ phonemes/km (95% confidence-level interval). b) Phonemic loss time equal to 20 generations/phoneme (i.e., 1 phoneme disappears every 20 generations in low-density population). For both regressions (blue and red solid lines) the simulated slope is -(1.7–1.8)·10^−3^ phonemes/km (95% confidence-level interval).

As mentioned above, in Fig 1 in Ref. [18] we fitted a straight line to the numbers of phonemes of present languages versus their distances from Atkinson’s most likely origin of the Out-of-Africa dispersal. We obtained the slope = -(3.4–6.5)·10^−4^ phonemes/km (95% confidence-level interval). This observed range and its mean correspond to the three green dash-dotted and dashed lines in Fig 1A and 1B in the present paper. Present-day African languages have a range of 35–40 phonemes (95% confidence level interval), as shown by the intercept of the upper and lower observed bounds (green dash-dotted and dashed lines) in Fig 1A and 1B. The first question we tackle is whether our model is consistent with this observed range, and if it is, for which values of the phonemic loss time.

Fig 1A and 1B show several examples of clines obtained from our simulations. Each figure includes two cases assuming, respectively, that the initial languages had 35–40 phonemes and 66–76 phonemes (the second case corresponds to click languages, as suggested by Fleming [46], see above). Thus, the squares and circles in Fig 1A and 1B correspond to possibilities (a) and (b), respectively, discussed in the previous section. Fig 1A and 1B display the simulated number of phonemes versus distance from the most likely origin of the Out-of-Africa dispersal, for two values of the phonemic loss time. Fig 1A shows a slow case with 80 generations per phoneme (i.e., one phoneme is randomly removed each 80 generations), and Fig 1B shows a fast case with 20 generations per phoneme (i.e., one phoneme is randomly removed each 20 generations). Note that, in each figure, the simulated slope is almost the same regardless of the initial number of phonemes (blue and red solid lines). Its value is -(4.3–4.9)·10^−4^ phonemes/km (95% confidence-level interval) for the slow case (80 generations/phoneme, Fig 1A), and -(1.7–1.8)·10^−3^ phonemes/km (95% confidence-level interval) for the fast case (20 generations/phoneme, Fig 1B).

First, we examine the initial conditions (a) in the Methods section i.e., an initial population in the central cell speaking a mix of languages with 35–40 phonemes (black squares in Fig 1A and 1B). For the slow case (80 generations per phoneme, Fig 1A), our model is able to properly simulate the observed phonemic cline, because almost all simulated languages lie within the upper and lower boundaries of the observed phonemic cline (green dash-dotted and dashed lines), and both the slope and the intercept agree very well with the observed values. In contrast, the fast loss time (20 generations per phoneme, Fig 1B) results in too strong an effect, in the sense that the simulated phonemic cline is much steeper than the observed one (green dash-dotted and dashed lines). This simulated cline, for the fast case and initial languages with 35–40 phonemes (Fig 1B), even vanishes well before the arrival of the pioneering populations at the end of the simulated domain (i.e., 25,000 km). Thus we have shown that, given the proper value of the phonemic loss time, our model is capable of reproducing the slope of the observed global phonemic cline. Clearly for initial languages with more phonemes (66–76 phonemes), the simulations (circles in Fig 1A and 1B) are above the observations (green lines), but remarkably, the slope from our model is essentially the same regardless of the range for the number of phonemes spoken by the initial populations (Fig 1A and 1B). Thus, the number of initial phonemes has virtually no impact on the slope of the simulated phonemic cline. Hence, it is by comparing slopes that we may infer differences in the type of processes (for comparison, in S1 Text, Sec I, we consider a different process with fast migrations, which leads to results inconsistent with the data). We would like to emphasize that none of the parameter values used in the model (net growth rate, dispersal distance, persistence, generation time and phonemic loss time) are derived from the dataset by Atkinson [11].

**Fig 2:**
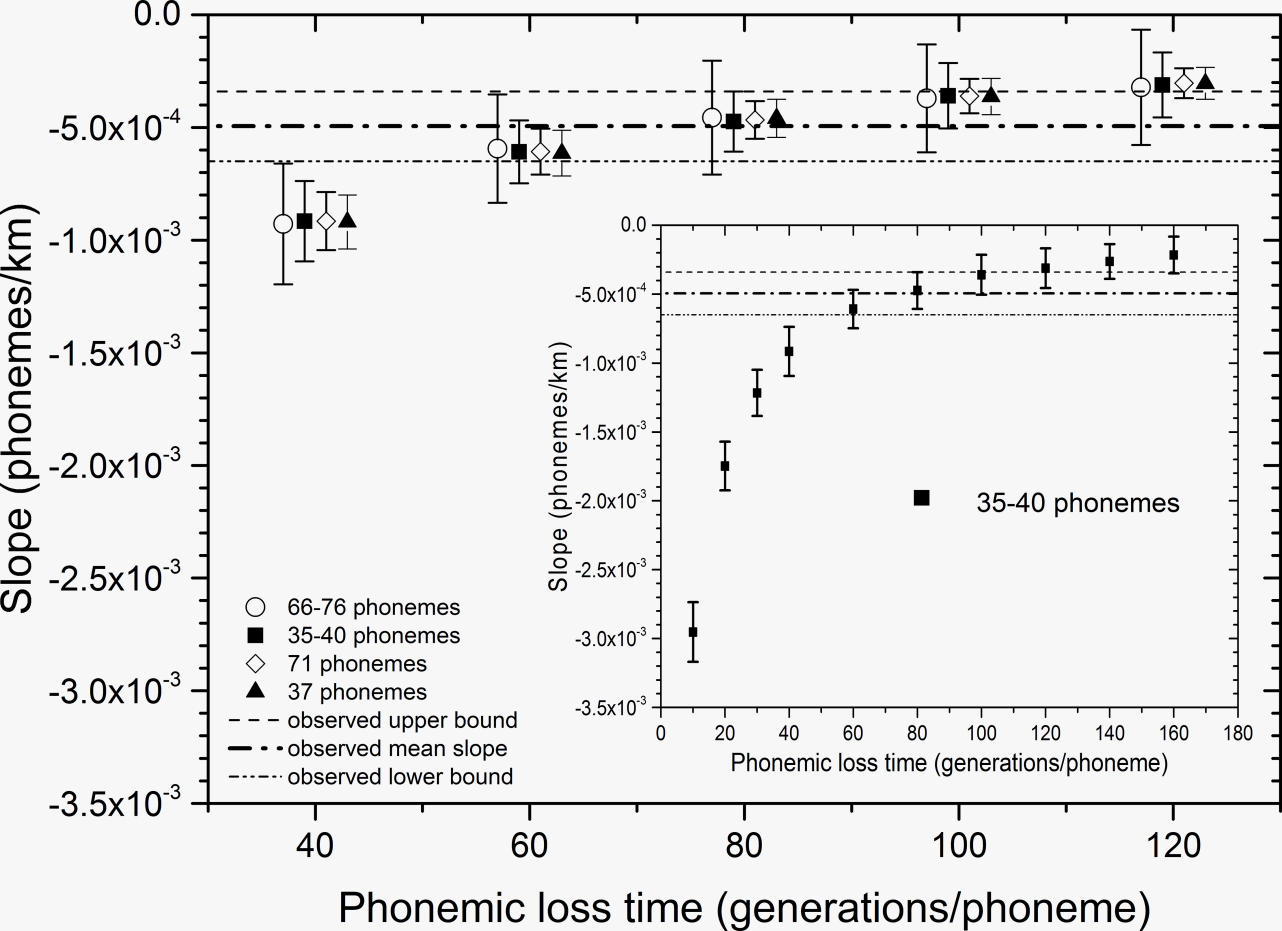
Plot of the slope of the linear fit to the simulated number of phonemes versus distance from the most likely origin of the Out-of-Africa dispersal, versus the time of phonemic loss (e.g., if the phonemic loss time is 80 generations/phoneme, then 1 phoneme disappears every 80 generations). In the main figure, results are shown for 40 to 120 generations/phoneme and four different initial populations. The insert shows, as an example, one initial population (speaking a mix of languages with 35–40 phonemes) over a wider range of phonemic loss times. Each error bar has been obtained from 100 numerical simulations, and represents the 95% confidence-level interval. At the start of the simulation only the central node of the grid is populated with a population of *N* = 10 tribes, speaking a randomly generated mix of synthetically generated languages within the given ranges of phonemes. The maximum number of tribes per node (*N*_*s*_) is set to 10 tribes/node and the demographic threshold below which phonemes are lost is also set to 10 tribes/node. Net fecundity *R*_*0*_ = 1.4. Time = 2,280 generations or about 70 kyr, corresponding to the present time from the Out-of-Africa dispersal. The dash-dotted and dotted lines give the mean, upper and lower bounds to the slope of the observed cline, as computed in Ref. [18], Fig 1, by performing a linear fit of the number of phonemes of 366 present-day languages versus their distances from the most likely origin of the Out-of-Africa dispersal (UPSID database). The result was [18] slope = -(3.4–6.5)·10^−4^ phonemes/km (95% confidence-level interval), *r* = -0.317, *p* < 0.001 (from Fig 1 in Ref. [18]).

Fig 2 shows, as error bars, the slopes of the linear fits to the simulated number of phonemes versus distance from Atkinson’s putative origin of the Out-of-Africa dispersal, as a function of the phonemic loss time. To estimate the effect of randomness (steps (i)-(iv) in Methods), each simulation has been repeated 100 times starting with the same initial conditions. The error bars in Fig 2 are due to the fact that two simulations with the same initial conditions do not yield exactly the same results. One reason for this is that, in the reproduction step, the languages that are reproduced are chosen at random (so in different simulation runs, they may have different numbers of phonemes). Another reason is that the languages that disperse are also chosen at random. A linear fit (such as those in Fig 1A and 1B) was performed for each simulation run, and the average and standard deviation (*σ*) of the slope were computed over 100 runs. The error bars give the range with 95% confidence level, i.e., ±2*σ* (we checked that the distribution of slopes is Gaussian, see S1 Text, Sec J). The observed range for the slope of the global phonemic cline, namely -(3.4–6.5)·10^−4^ phonemes/km, is shown in Fig 2 as horizontal dash-dotted and dotted lines (95% confidence intervals from Fig 1 in Ref. [18]). Because we do not know the number of phonemes of the language(s) spoken at the onset of the out of Africa dispersal, we consider several possibilities and show that they yield similar results. Fig 2 displays results for initial languages with 35–40 phonemes (possibility (a) in Methods), 66–76 phonemes (possibility (b) in Methods), as well as for a single initial language with 37 (possibility (a)) and 71 phonemes (again possibility (b)). The range of total phoneme numbers tested is wide, reflecting the uncertainty of our present knowledge about archaic human language(s). We can see that the simulated slopes follow the same trend, regardless of the phonemic composition of the initial set of languages. Therefore, the slope of the simulated cline is insensitive to the number of phonemes and mixture of languages at the onset of the Out-of-Africa dispersal. Any phonemic loss time within the range of 60 to 120 generations/phoneme yields simulated slopes within the observed range. In the insert to Fig 2 we see that any value for the phonemic loss time smaller than 50 generations/phoneme produces slopes that are inconsistent with the observed phonemic cline. The fact that the slopes of the simulated clines fall within the range of the observed global phonemic cline, for different initial conditions (Fig 2), is encouraging. We can conclude that, regardless the phonemic diversity and mixture of languages at the onset of the dispersal, and for an appropriate range of phonemic loss time, our simulations can reproduce the slope of the observed cline (Fig 2).

### 3.2 Cline for the diversity *t*_*F*_ of phonemes

In the previous sections, we have focused our research on the total number of phonemes, because Atkinson detected a cline for it [11] and suggested a SFE as a possible explanation. We have seen that, for a cline similar to the observed one to be generated, a SFE is not enough. It is also necessary that either low-density populations lose phonemes (present paper) or that high-density populations gain phonemes [18]. In contrast, the worldwide observed cline of genetic diversity could have been generated simply by a SFE, i.e., by a repeated loss of diversity in populations (because of fissions or extinctions of parts of them) [13,14]. On the other hand, it is important to note that diversity is not a measure of the number of traits (richness). It is rather a measure of the evenness characterizing the distribution of traits across classes [75–77]. Indeed, one of the criticisms of the work by Atkinson was that he did not distinguish between richness (number of phonemes) and diversity (evenness in the distribution of phonemes) [25]. Then the question arises whether, similarly to the observed cline for the number of phonemes [11], phonemic diversity of languages also shows a cline from the putative origin, and if it does, whether it could be also attributed to the Out-of-Africa dispersal. Here we use the statistic *t*_*F*_ as a measure of the diversity in phonemes (or other cultural traits) [75],

$$t_{F}=\frac{1}{\sum_{i=1}^{nph}p_{i}^{2}}-1$$ (1)

where *p*_*i*_ is the relative frequency of phoneme (*i* = 1,2,3,…,*nph*), obtained by dividing the number of times of occurrence of phoneme *i* over all languages by the sum of these numbers over all phonemes. *nph* = 908 is the number of phonemes in our database (see below).

It is important to note that the cline of the number of phonemes does not univocally determine the cline of phonemic diversity *t*_*F*_. The reason is that the number of phonemes does not fix the value of *t*_*F*_. For example, consider the very simple case of a set of languages with only two phonemes. If their frequencies are *p*_1_ = *p*_2_ = 0.5, the Eq (1) implies *t*_*F*_ = 1, but if e.g., *p*_1_ = 0.75 and *p*_2_ = 0.25 then Eq (1) yields *t*_*F*_ = 0.6 (and the distribution is less even or less diverse). Thus, the existence (or not) of a cline for the diversity *t*_*F*_ has to be analyzed independently of that of a cline for the number of phonemes.

To calculate the phonemic diversity of a set of languages, we need their phonemic inventories. We could find the list of phonemes for 359 languages (S1 Database). In these 359 languages, we found 908 different phonemes. First, all the languages in our dataset were coded into strings of “1”s and “0”s. This lead to a ‘complete’ matrix of 359 rows (languages) x 908 columns (phonemes). The presence of a phoneme is marked with a “1” in the corresponding position. The absence of a given phoneme is marked with a “0”.

At the beginning of each simulation run, the subset of the complete matrix with only the phonemes of the initial languages (used in the specific simulation run) is stored. For example, if initially there are *N* = 10 tribes, the ‘initial’ matrix has 10 rows and 908 columns with the information (presence/absence) of phonemes. At the end of each row, we also add a column with a letter that will be used to univocally identify each of the 10 languages (in the specific simulation run).

Next we note that any given language can be transformed into a string of consecutive “1”s followed by a string of consecutive “0”s, plus the letter that identifies the language (in the simulation run considered). This ‘transformed’ initial matrix is used in our simulations, and it substantially speeds up the computing time (because it has 94+1 columns, whereas the initial, untransformed matrix has 908+1 columns, due to the fact that in our database there are 908 different phonemes, but in our simulations there are no initial languages with more than 94 phonemes, and 95 digits is the maximum row length allowed by our FORTRAN compiler).

During the simulation, the transformed initial matrix evolves in the following way. Phonemes are randomly removed from languages (i.e., a randomly chosen position occupied by a “1” is converted into a “0”) at every interval set by the prescribed phonemic loss time (for example, if the phonemic loss time is 80 generations/phoneme, then one phoneme is removed every 80 generations). Note that, as a result, after some point during the simulation, some languages will have “0”s in between the series of “1”s.

At the end of the simulation, we need to know to which of the 908 phonemes each of the 94 “1”s or “0”s corresponds. In other words, we need to transform each row of 94 “1”s or “0”s into a row of 908 “1”s or “0”s. In order to do so, we use the letter at the end of each row to retrieve the initial sequence of 908 “1”s or “0”s (from the initial untransformed matrix). Then we convert the final list of 94 “1”s or “0”s into a list of 908 “1”s or “0”s, as follows: all “0”s in the initial list of 908 “1”s or “0”s will still be “0”s in the final list, but some “1”s will have mutated into “0”s. For example, if position 10 in the initial list of 94 digits was a “1” and it is a “0” in the final list of 94 digits, then the tenth “1” in the initial list of 908 digits will be a “0” in the final list of 908 digits. In this way, we can find the complete lists of 908 “1”s or “0”s for all final languages, and use them to compute the frequencies *p*_*i*_ of all phonemes over these languages, which finally are used to calculate the value of the statistic *t*_*F*_ using Eq (1).

**Fig 3:**
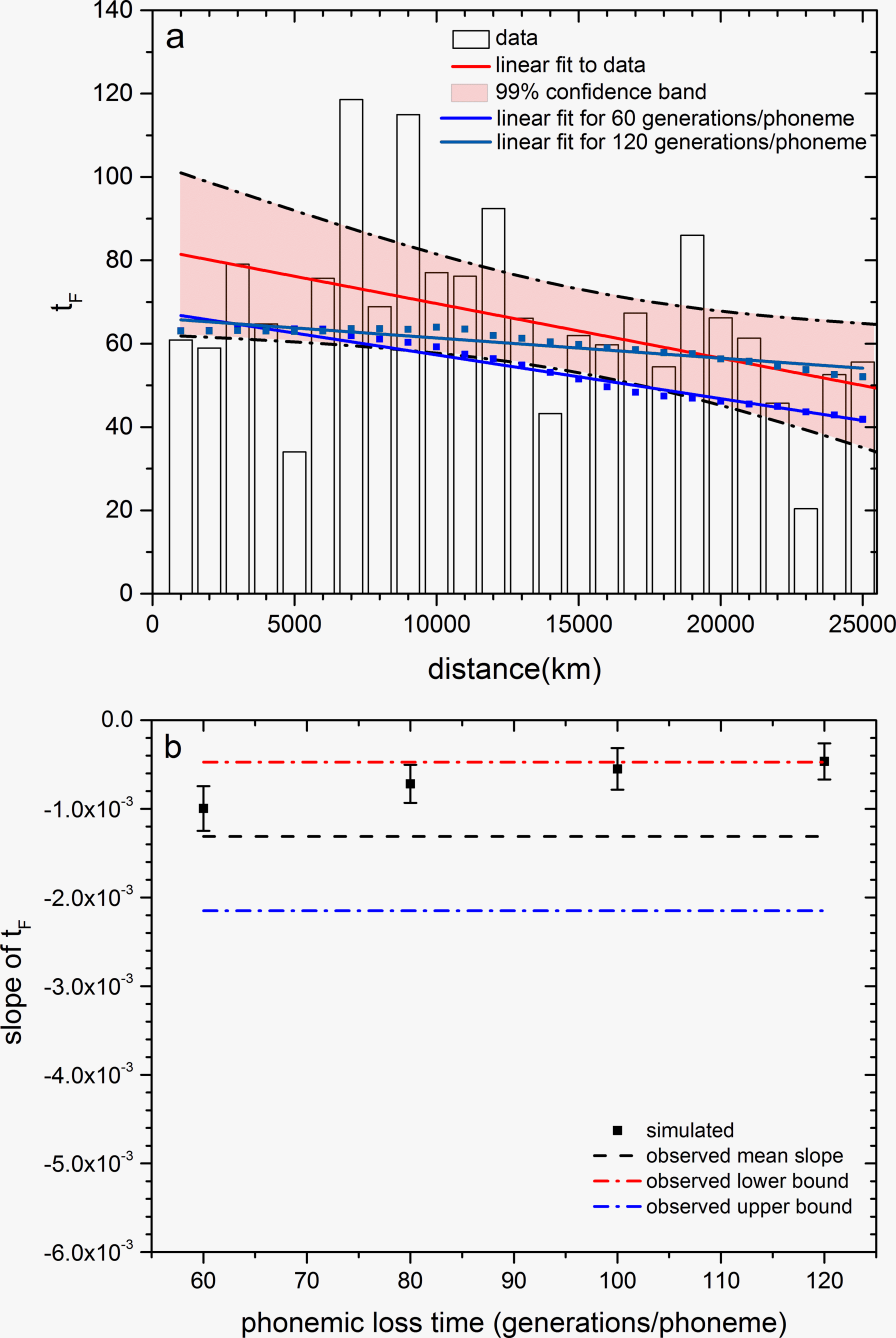
Results for the phonemic diversity *t*_*F*_. At the start of the simulation, only the central node of the grid is populated with a population of *N* = 10 tribes, speaking a mix of two languages (5 tribes speak Ewe and 5 speak Ngizim) with 40 phonemes each. The maximum number of tribes per node (*N*_*s*_) is set to 10 tribes/node and the demographic threshold below which phonemes are lost is also set to 10 tribes/node. Net fecundity *R*_*0*_ = 1.2. Time = 2,280 generations or about 70 kyr, corresponding to the present time from the Out-of-Africa dispersal. a) The blue and green squares are the simulation results for the phonemic diversity (measured as *t*_*F*_) versus distance, computed over simulated languages binned in 1000-km intervals for two phonemic loss times, namely 60 generations/phoneme (blue line, slope = -(0.9–1.1)·10^−3^ km^-1^, *r* = -0.983) and 120 generations/phoneme (green line, slope = -(0.4–0.57)·10^−3^ km^-1^, *r* = -0.922). The observed phonemic diversity cline (measured as *t*_*F*_) is also shown (histogram, see S1 Text, Sec A for details). The linear fit to the observed histogram (red line) has slope = -(0.5–2.1)·10^−3^ km^-1^ (all slopes are reported with 95% confidence level). b) Dependence of the slope of the linear fit to the simulated phonemic diversity cline on the time of phonemic loss (e.g., if the phonemic loss time is 80 generations/phoneme, then 1 phoneme disappears every 80 generations). Each error bar has been obtained from 100 numerical simulations, and shows the 95% confidence-level interval. The dotted and dash-dotted lines give the mean, upper and lower bounds to the slope of the observed phonemic diversity cline (obtained from Fig 3A).

Before running the simulations, we checked that there is a global phonemic diversity cline in present human languages. In Fig 3A, each value of *t*_*F*_ (vertical bar) has been computed using all languages in our database whose location is within a 1000 km interval. The slope does not change appreciably if we compute *t*_*F*_ by grouping languages using other distance intervals, e.g. 1500 km, 2000 km and 3000 km (see S1 Text, Sec A). The FORTRAN code used to compute the value of *t*_*F*_ for languages within a given distance interval is provided as a separate file in S2 Software.

We next tackle the question of whether our model can generate the observed global phonemic cline of diversity *t*_*F*_, and under which conditions.

In Fig 3A we include two simulated clines (squares and regression lines) of phonemic diversity *t*_*F*_, which are consistent with the observed one. They have been obtained using two initial languages (with 40 phonemes each, not all of them the same), a net fecundity of *R*_*0*_ = 1.2, and two values of the phonemic loss time (60 and 120 generations/phoneme). Other values of *R*_*0*_ (e.g. *R*_*0*_ = 1.3) also yield clines of *t*_*F*_ consistent with the observed one, but only if the net fecundity is within the range 1.1 < *R*_*0*_ < 1.4. We have checked that these simulations also yield a cline for the number of phonemes (instead of the diversity *t*_*F*_) that is consistent with the cline detected by Atkinson for the number of phonemes (S1 Text, Sec B). The reason why the phonemic diversity cline disappears if *R*_*0*_ ≥ 1.4 is that then, cells at the forefront reach saturation very quickly, thus inhibiting the reduction of diversity (because phonemes disappear only if the population density is low enough). On the other hand, if *R*_*0*_ ≤ 1.1, the simulated cline of *t*_*F*_ still appears but the profile of the population density has an oscillating shape, which is not acceptable (see S1 Text, Sec C for a detailed explanation).

We also tackled the question of whether there is a minimum number of initial languages that can reproduce the observed global phonemic diversity cline. We found that the answer is negative, i.e., that a cline of *t*_*F*_ consistent with the obtained one can be obtained regardless of the number of languages spoken at the onset of the Out-of-Africa dispersal (S1 Text, Sec D).

Fig 3B shows the dependence of the slope of the simulated phonemic diversity cline versus the time of phonemic loss (e.g., if the phonemic loss time is 80 generations/phoneme, then 1 phoneme disappears every 80 generations). Each error bar has been obtained from 100 numerical simulations, and represents the 95% confidence-level interval. The dashed and dash-dotted lines give the mean, upper and lower bounds to the slope of the observed phonemic diversity cline as obtained in Fig 3A, namely slope = -(0.5–2.1)·10^−3^, *r* = -0.509 (see the caption of Fig 3). In Fig 3B, we see that the simulations yield clines of diversity *t*_*F*_ consistent with the observed one if the phonemic loss time is less than 120 generation/phoneme. We have not considered phonemic loss times below 60 generations/phoneme, because they do not yield a cline for the number of phonemes consistent with the observed one (Fig 2). Note that in Fig 3B, the magnitude of the slope of *t*_*F*_ is smaller the longer the phonemic loss time, corresponding to a slower (and thus less important) reduction in phonemes.

It is worth noting that simulations of the phonemic diversity cline do not show the abrupt peaks observed in the plot of phonemic diversity obtained from observed data (Fig 3A). One reason is that data on extant languages are not equally distributed across the intervals, whereas for the simulations the number of languages per bin is always the same (for a 1000-km bin, there are 20 cells and 10 languages/cell, i.e., 200 languages). Another reason is that our model does not account for other mechanisms that produced diversity, such as Neolithic migrations, other population movements and linguistic diversification after the Out-of-Africa dispersal.

Finally, we have also used our simulations to explore the time needed for a SFE signal to disappear (S1 Text, Sec E). We have found that, even for a deep temporal scale of 300 kyr, and in the absence of other processes that may have eroded the original signal, the clines for the number of phonemes and the phonemic diversity *t*_*F*_ are still clearly seen. This is at variance with some claims that any SFE signal would quickly disappear [29,34,39,41,42], which in contrast to the present paper are not supported by numerical simulations.

## 4. Conclusions

We have performed simulations assuming that low-density populations lose phonemes. Atkinson [11] proposed that this could generate a cline of phonemic inventory size, but he did not test his idea using numerical simulations. In contrast, we have obtained quantitatively a cline consistent with the observed one (not only for the number of phonemes but also for the diversity *t*_*F*_). We have not assumed copying errors [15], Darwinian competition [16], contrastive possibilities [17], nor any other specific phonemic loss mechanism. In this sense, a cline arises regardless of the precise mechanism of phonemic evolution.

The results suggest some interesting conclusions. Our simulations indicate that a phonemic serial founder effect, taking place during the Out-of-Africa dispersal, can reproduce the global phonemic cline, not only for the number of phonemes but also for the phonemic diversity (here measured as *t*_*F*_). We would like to stress, however, that these conclusions are based on two assumptions, namely: (i) languages at the onset of the Out-of-Africa dispersal had high phonemic inventories (similar to the present languages in Africa); and (ii) a phonemic loss process for languages with low numbers of speakers per unit area (as assumed by Atkinson). Both assumptions have some justification. Concerning assumption (i), archaeology [49] and genetics [13,14] both support the idea of a higher human diversity in Africa than in the rest of the world. And assumption (ii), i.e., a higher probability of losing information in smaller groups, has also been proven many times in cultural evolution research, both empirically [52–54] and theoretically [75,77]. This simple exercise highlights the power of quantitative models for gaining insights from an hypothesis expressed with natural language.

The present paper provides an alternative to a previous model that also reproduced the observed cline [18], but was not based on Atkinson’s proposal. In the new model, the observed slope can be reproduced for a sufficiently long phonemic loss time (Fig 2, inset). On the other hand, initial languages spoken at the onset of the Out-of-Africa dispersal cannot lead to the observed cline if they had many phonemes, similarly to present-day click languages (Fig 1, open circles). However, it is worth stressing that the number of phonemes is irrelevant when determining the slope (Fig 1A and 1B). The reason is that the phonemic loss process acts on low-density populations, but the population density is independent on the number of languages. Low-density populations occur at the leading edge of the wave of advance (pioneering populations during the Out-of-Africa dispersal). This process of phonemic loss takes place repeatedly as the front advances, so its effect is greater at longer distances, and this causes the clines (both in the number of phonemes and their diversity). Thus, the slope of the cline is not affected by the initial number of phonemes.

It is worth to note that, taking into account evidence of modern behavior and fossil evidence outside Africa [78–80], it can be assumed that language was already widespread in Africa and Asia about 120 kyr. Then we would have to consider at least 50 kyr of linguistic evolution (possibly including phonemic accumulation [44], [18]) until the out-of-Africa dispersal 70 kyr ago. This is not inconsistent with the present paper, because isolated and/or low-density languages would not have accumulated so many phonemes. Thus, in such a framework, the initial languages (with few phonemes) in the simulations reported in the present paper would have been present in some regions (due to an isolation or fission event), but in other regions of Africa the number of phonemes could have been significantly higher. This means that a combination of phonemic accumulation in high-density populations (as proposed in Ref. [18]) and phonemic loss in low-density populations (as proposed in the present paper) would be viable. Interestingly, it would be similar to the fast rates of word gain in large populations and fast rates of word loss in low-density populations, which have been estimated from observed data in Ref. [54].

Our simulation models can be applied in principle to any set of cultural traits (not necessarily phonemes), provided that they are not strongly affected by selection. On the other hand, there is a tradeoff between the complexity of a model and the uncertainty added at the expense of having a better fit to the data [81]. A more complex model, lacking in data of high quality to calibrate and validate it, might yield unrealistic results. The more parameters added to the model, the better the calibration, until a perfect match between the simulated and the observed data can be obtained. But the uncertainty introduced in these more complex models may render their results useless (i.e., the model may be overfitted). Our model explores the hypothesis that a SFE could generate the observed global phonemic cline and we have used the fewest parameters necessary. Except for the phonemic loss time and the initial phoneme inventory sizes, which we cannot estimate directly, all other parameter values in our model have been estimated from ethnographic and/or archaeological data obtained from observations independent of the phonemic cline. It is true that we have had to choose the initial languages in our simulation runs, but this does not affect the conclusions (S1 Text, Sec D).

Future work, replacing our simple homogeneous dispersal kernel with a more realistic kernel that takes into account landmasses (oceans, mountain ranges, etc.), would refine the simulated phonemic and diversity clines. Also, the model can be scaled down to study patterns of phonemic diversity at a regional level. Migration routes could be also implemented to study hypothetical dispersal scenarios.

Finally, we stress that the modelling exercise reported in the present paper provides a quantitative approach to the proposal by Atkinson [11] that the worldwide phonemic cline detected by him could be due to low-density populations losing phonemes. It is also important to emphasize that our previous model (in which high-density populations gain phonemes [18]) and the new model in this paper (in which low-density populations lose phonemes) can both explain the cline in the number of phonemes detected by Atkison [11]. Then the question arises whether it is possible or not to determine which one of either models (or even a combination of them), if any, is valid. Future work could contribute to this question in two directions. Firstly, it may be possible in principle to detect more features in the observed data (in addition to the cline in the number of phonemes [11]) and determine whether both models yield different predictions for them or not (the cline of phonemic diversity *t*_*F*_ considered here for the first time is one example, but in principle more regularities could perhaps be found in the spatial distribution of present languages). Secondly, perhaps it will be possible to detect empirically whether low-density populations lose phonemes and/or high-density populations gain them. Indeed, this seems the only possible way to show conclusively whether our models are valid or not. Note, for example, that Fig 2 in the present paper predicts a range for the phonemic loss time (60–120 generations/phoneme) that makes our new model consistent with the observed cline. If the phonemic loss time could be measured in real low-density populations, it should fall within this range (for the model in this paper to be valid). It is encouraging that it has been shown empirically that smaller populations have higher rates of word loss, and that larger populations have higher rates of gain of new words [54]. If a similar effect could be measured empirically for phonemes instead of words, it would advance the problem tackled in the present paper tremendously.

## Supporting information

S1 Text Supplementary results. (DOCX)

S1 Database Language database. (XLSX)

S1 Software SFE with phonemic loss program in FORTRAN. (ZIP)

S2 Software Program to compute diversity *t*_*F*_ of languages at given distance intervals in FORTRAN. (ZIP)

## References

[1] Ruhlen M. The origin of language: tracing the evolution of the mother tongue 1st ed. Wiley; 1994.

[2] Coupé C, Hombert J-M. Polygenesis of linguistic strategies: a scenario for the emergence of language In: Minett J, Shi-Yuan Wang W, editors. Language acquisition, change and emergence: essays in evolutionary linguistics, Chapter: 3. Hong Kong: City University of Hong Kong Press; 2005 pp. 153–201.

[3] Corballis MC. From hand to mouth The origins of language. 1 st ed Princeton, NJ: Princeton University Press; 2002.

[4] Pagel M. Darwinian perspectives on the evolution of human languages. Psychonomic Bulletin & Review. 2017; 24(1): 151–157. 27368626 10.3758/s13423-016-1072-z PMC5325856

[5] Bromham L. Curiously the same: swapping tools between linguistics and evolutionary biology. Biol Philos. 2017; 32: 855–886.

[6] Pakendorf B. Coevolution of languages and genes. Current Opinion in Genetics & Development. 2014; 29: 39–44. 25170984 10.1016/j.gde.2014.07.006

[7] Oppenheimer S. Out-of-Africa, the peopling of continents and islands: tracing uniparental gene trees across the map. Phil Trans R Soc B. 2012; 367: 770–784. doi: 10.1098/rstb.2011.0306 22312044 10.1098/rstb.2011.0306 PMC3267120

[8] Stewart JR, Stringer CB. Human evolution out of Africa: the role of refugia and climate change. Science 335; 2012: 1317–1321. doi: 10.1126/science.1215627 22422974 10.1126/science.1215627

[9] Nichols J. Monogenesis or polygenesis: a single ancestral language for all humanity? In: Gibson KR, Tallerman M, editors. The Oxford handbook of language evolution. Oxford, UK: Oxford University Press; 2013 pp. 559–572.

[10] Gray RD. Evolution. Pushing the time barrier in the quest for language roots. Science. 2005; 309: 2007–2008. doi: 10.1126/science.1119276 16179464 10.1126/science.1119276

[11] Atkinson QD. Phonemic diversity supports a serial founder effect model of language expansion from Africa. Science. 2011; 332, 346–349. doi: 10.1126/science.1199295 21493858 10.1126/science.1199295

[12] Kacprzak S, Ziółko M, Masior M, Igras M, Ruszkiewicz M. Statistical analysis of phonemic diversity in languages across the world. Proceedings of the XIX national conference applications of mathematics in biology and medicine. 2013 Sep 16–20; Jastrzębia Góra, Poland. ISBN:978-83-60253-86-1. pp. 48–53.

[13] Ramachandran S, Deshpande O, Roseman CC, Rosenberg NA, Feldman MW, Cavalli-Sforza LL. Support from the relationship of genetic and geographic distance in human populations for a serial founder effect originating in Africa. PNAS. 2005; 102: 15942–15947. doi: 10.1073/pnas.0507611102 16243969 10.1073/pnas.0507611102 PMC1276087

[14] Deshpande O, Batzoglou S, Feldman MW, Cavalli-Sforza LL. A serial founder effect model for human settlement out of Africa. Proc R Soc B. 2009; 276: 291–300. doi: 10.1098/rspb.2008.0750 18796400 10.1098/rspb.2008.0750 PMC2674957

[15] De Boer B. Self-organization in vowel systems. Journal of Phonetics. 2000; 28: 441–465.

[16] Ritt N. Selfish sounds and linguistic evolution: a Darwinian approach to language change 1st ed Cambridge, UK: Cambridge University Press; 2004.

[17] Trudgill P. Social structure and phoneme inventories. Linguistic Typology. 2011; 15(2): 155–160.

[18] Fort J, Pérez-Losada J. Can a linguistic serial founder effect originating in Africa explain the worldwide phonemic cline? J R Soc Interface. 2016; 13: 20160185, 1–9. 10.1098/rsif.2016.0185 PMC4874439 27122180

[19] Henn BM, Cavalli-Sforza LL, Feldman MW. The great human expansion. PNAS. 2012; 109: 17758–17764. doi: 10.1073/pnas.1212380109 23077256 10.1073/pnas.1212380109 PMC3497766

[20] Hay J, Bauer L. Phoneme inventory size and population size. Language. 2007; 83: 388–400.

[21] Donohue M, Nichols J. Does phoneme inventory size correlate with population size? Linguistic Typology. 2011; 15(2): 161–170.

[22] Vaesen K, Collard M, Cosgrove R, Roebroeks W. Population size does not explain past changes in cultural complexity. PNAS. 2016; 113(16): E2241–E2247. doi: 10.1073/pnas.1520288113 27044082 10.1073/pnas.1520288113 PMC4843435

[23] Henrich J, Boyd R, Derex M, Kline MA, Mesoudi A, Muthukrishna M, et al Understanding cumulative cultural evolution. PNAS. 2016; 113 (44): E6724–E6725. doi: 10.1073/pnas.1610005113 27791123 10.1073/pnas.1610005113 PMC5098628

[24] Vaesen K, Collard M, Cosgrove R, Roebroeks W. Reply to Henrich et al.: The Tasmanian effect and other red herrings. PNAS. 2016; 113(44): E6726–E6727. doi: 10.1073/pnas.1613074113 27791150 10.1073/pnas.1613074113 PMC5098611

[25] Maddieson I, Bhattacharya T, Smith EE, Croft W. Geographical distribution of phonological complexity. Linguistic Typology. 2011; 15(2): 267–280.

[26] Dryer MS, Martin H (editors). 2013 [accessed on 2014-05-16]; The world atlas of language structures online Leipzig: Max Planck Institute for Evolutionary Anthropology Available from: http://wals.info.

[27] Cysouw M, Dediu D, Moran S. Comment on “Phonemic diversity supports a serial founder effect model of language expansion from Africa”. Science. 2012; 335(6069): 657. 10.1126/science.1208841 22323802

[28] Maddieson I, Precoda K. Updating UPSID. UCLA working papers in Phonetics. 2011; 74: 104–111.

[29] Sproat R. Phonemic diversity and the out-of-Africa theory. Linguistic Typology. 2011; 15(2): 199–206.

[30] Atkinson QD. Linking spatial patterns of language variation to ancient demography and population migrations. Linguistic Typology. 2011; 15(2): 321–332.

[31] Atkinson QD. Response to comments on ''Phonemic diversity supports a serial founder effect model of language expansion from Africa''. Science. 2012; 335(6069): 657. 10.1126/science.1207846 22323803

[32] Trudgill P. Linguistic and social typology: The Austronesian migrations and phoneme inventories. Linguistic Typology. 2004; 8(3): 305–320.

[33] Pericliev V. On phonemic diversity and the origin of language in Africa. Linguistic Typology. 2011; 15(2): 217–222.

[34] Bowern C. Out of Africa? The logic of phoneme inventories and founder effects. Linguistic Typology. 2011; 15(2): 207–216.

[35] Wang CC, Ding QL, Tao H, Li H. Comment on “Phonemic diversity supports a serial founder effect model of language expansion from Africa”. Science. 2012; 335–657. 10.1126/science.1208841 22323802

[36] Munroe RL, Fought JG, Macaulay RKS. Warm climates and sonority classes: Not simply more vowels and fewer consonants. Cross-Cultural Research. 2009; 43: 123–133.

[37] Greenhill SJ. Overview: Debating the effect of environment on language. Journal of Language Evolution. 2016; 1(1): 30–32.

[38] Coupé C, Hombert J-M, Marisco E, Pellegrino F. Investigations into determinants of the diversity of the world’s languages In: Gang P, Feng S, editors. Eastward flows the great river. Hong Kong: City University of Hong Kong Press; 2013 pp. 75–108.

[39] Bybee J. How plausible is the hypothesis that population size and dispersal are related to phoneme inventory size? Linguistic Typology. 2011; 15(2): 147–154.

[40] Van Tuyl R, Pereltsvaig A. Comment on “Phonemic diversity supports a serial founder effect model of language expansion from Africa”. Science. 2012; 335(6069):657. 10.1126/science.1209176 22323804

[41] Hunley K, Bowern C, Healy M. Rejection of a serial founder effects model of genetic and linguistic evolution. Proc R Soc B. 2012; 279: 2281–2288. doi: 10.1098/rspb.2011.2296 22298843 10.1098/rspb.2011.2296 PMC3321699

[42] Dahl Ö. Are small languages more or less complex than big ones? Linguistic Typology. 2011; 15(2): 171–175.

[43] Handley LJ, Manica A, Goudet J, Balloux F. Going the distance: human population genetics in a clinal world. Trends Genet. 2007; 23(9): 432–439. doi: 10.1016/j.tig.2007.07.002 17655965 10.1016/j.tig.2007.07.002

[44] Perrault C, Mathew S. Dating the origin of language using phonemic diversity. PLoS One. 2012; 7: e35289 doi: 10.1371/journal.pone.0035289 22558135 10.1371/journal.pone.0035289 PMC3338724

[45] Ruhlen M. A Guide to the world’s languages Vol. 1 1st ed. Stanford, CA: Stanford University Press; 1991.

[46] Fleming L. Phoneme inventory size and the transition from monoplanar to dually patterned speech. Journal of Language Evolution, Special Issue: SI. 2017; 2(1): 52–66.

[47] Brugman, JC. Segments, tones and distribution in Khoekhoe prosody. Ph.D. Thesis, Cornell University. 2009. Available from: https://ecommons.cornell.edu/bitstream/handle/1813/13921/Brugman%2C%20Johanna.pdf?sequence=1&isAllowed=y

[48] Ringe D. A pilot study for an investigation into Atkinson’s hypothesis. Linguistic Typology. 2011; 15(2): 223–231.

[49] Romanowska I, Gamble C, Bullock S, Sturt F. Dispersal and the Movius line: testing the effect of dispersal on population density through simulation. Quaternary International. 2017; 431(Part B): 53–63.

[50] Powell A, Shennan S, Thomas MG. Late Pleistocene demography and the appearance of modern human behavior. Science. 2009; 324: 1298–1301. doi: 10.1126/science.1170165 19498164 10.1126/science.1170165

[51] Johnson AW, Earle TK. The evolution of human societies: from foraging group to agrarian state 1st ed. Stanford, CA: Stanford University Press; 1987.

[52] Collard M, Ruttle A, Buchanan B, O’Brien MJ. Population size and cultural evolution in nonindustrial food-producing societies. PLoS One. 2013; 8(9): e72628 doi: 10.1371/journal.pone.0072628 24069153 10.1371/journal.pone.0072628 PMC3772076

[53] Derex M, Beugin M-P, Godelle B, Raymond M. Experimental evidence for influence of group size on cultural complexity. Nature. 2013; 503: 389–391. doi: 10.1038/nature12774 24226775 10.1038/nature12774

[54] Bromham L, Hua X, Fitzpatrick TG, Greenhill SJ. Rate of language evolution is affected by population size. PNAS. 2015; 112: 2097–2102. doi: 10.1073/pnas.1419704112 25646448 10.1073/pnas.1419704112 PMC4343108

[55] Wichmann S, Rama T, Holman EW. Phonological diversity, word length, and population sizes across languages: the ASJP evidence. Linguistic Typology. 2011; 15(2): 177–198.

[56] Creanza N, Ruhlen M, Pemberton TJ, Rosenberg NA, Feldman MW, Ramachandran S. A comparison of worldwide phonemic and genetic variation in human populations. PNAS. 2015; 112: 1265–1272. doi: 10.1073/pnas.1424033112 25605893 10.1073/pnas.1424033112 PMC4321277

[57] Hunley K. Reassessment of global gene-language coevolution. PNAS. 2015; 112 (7): 1919–1920. doi: 10.1073/pnas.1425000112 25675520 10.1073/pnas.1425000112 PMC4343119

[58] Reyes-Centeno H. Out of Africa and into Asia: fossil and genetic evidence on modern human origins and dispersals. Quaternary International. 2016; 416: 249–262.

[59] Crow JF, Kimura M. An introduction to population genetics theory 1st ed. New York: Harper & Row; 1970.

[60] Reyes-Centeno H, Harvati K, Jäger G. Tracking modern human population history from linguistic and cranial phenotype. Scientific Reports. 2016; 6: 36645 doi: 10.1038/srep36645 27833101 10.1038/srep36645 PMC5105118

[61] Dyen I, Kruskal JB, Black P. An Indoeuropean classification: a lexicostatistical experiment. Transactions of the American Philosophical Society. 1992; 82(5): 1–132.

[62] Fort J, Pérez-Losada J, Isern N. Fronts from integrodifference equations and persistence effects on the Neolithic transition. Phys Rev E. 2007; 76(3): 031913. 10.1103/PhysRevE.76.031913 17930277

[63] Hassan FA. Demographic archaeology New York: Academic Press; 1981. Table 2.1.

[64] Dixon RMW. Tribes, languages and other boundaries in northeast Queensland In: Peterson N, editor. Tribes and boundaries in Australia. Canberra: Australian institute of aboriginal studies; 1976 pp. 207–238.

[65] Fort J, Jana D, Humet J. Multidelayed random walks: theory and application in Neolithic transition in Europe. Phys Rev E. 2004; 70(3): 031913. 10.1103/PhysRevE.70.031913 15524555

[66] Davison K, Dolukhanov P, Sarson GR, Shukurov A. The role of waterways in the spread of the Neolithic. Journal of Archaeological Science. 2006; 33: 641–652.

[67] Fort J, Pujol T. Progress in front propagation research. Rep Prog Phys. 2008; 71(8): 086001.

[68] Fort J. Synthesis between demic and cultural diffusion in the Neolithic transition in Europe. PNAS. 2012; 109(46): 18669–18673. doi: 10.1073/pnas.1200662109 23112147 10.1073/pnas.1200662109 PMC3503213

[69] Pérez-Losada J, Fort J. Spatial dimensions increase the effect of cultural drift. Journal of Archaeological Science. 2011; 38: 1294–1299.

[70] Conolly J, Colledge S, Shennan S. Founder effect, drift, and adaptive change in domestic crop use in early Neolithic Europe. J Arch Sci. 2008; 35: 2797–2804.

[71] Bentley RA, Hahn MW, Shennan S. Random drift and culture change. Proc R Soc B. 2004; 271(1547):1443–1450. doi: 10.1098/rspb.2004.2746 15306315 10.1098/rspb.2004.2746 PMC1691747

[72] Reali F, Griffiths TL. Words as alleles: connecting language evolution with Bayesian learners to models of genetic drift. Proc R Soc B. 2010; 277: 429–436. doi: 10.1098/rspb.2009.1513 19812077 10.1098/rspb.2009.1513 PMC2842651

[73] Petraglia MD, Haslam M, Fuller DQ, Boivin N, Clarkson C. Out of Africa: new hypotheses and evidence for the dispersal of *Homo sapiens* along the Indian Ocean rim. Ann Hum Biol. 2010; 37: 288–311. doi: 10.3109/03014461003639249 20334598 10.3109/03014461003639249

[74] O’Connell JF, Allen J. The restaurant at the end of the universe: modelling the colonisation of Sahul. Australian Archaeology. 2012; 74: 5–17.

[75] Neiman FD. Stylistic variation in evolutionary perspective: inferences from decorative diversity and interassemblage distance in Illinois woodland ceramic assemblages. American Antiquity. 1995; 60(1): 7–36.

[76] Nettle Daniel, Linguistic diversity 1st ed Oxford, UK: Oxford University Press; 1999.

[77] Shennan S, Wilkinson JR. Ceramic style change and neutral evolution: a case study from Neolithic Europe. American Antiquity. 2001; 66: 577–594.

[78] Groucutt HS, Petraglia MD, Bailey G, Scerri EML, Parton A, Clark-Balzan L, et al Rethinking the dispersal of *Homo sapiens* out of Africa. Evolutionary Anthropology: Issues, News, and Reviews. 2015; 24(4): 149–64. 10.1002/evan.21455 PMC6715448 26267436

[79] Grove M, Lamb H, Roberts H, Davies S, Marshall M, Bates R, et al Climatic variability, plasticity, and dispersal: A case study from Lake Tana, Ethiopia. J Hum Evol. 2015; 87: 32–47. doi: 10.1016/j.jhevol.2015.07.007 26472274 10.1016/j.jhevol.2015.07.007

[80] Liu W, Martinón-Torres M, Cai Y-J, Xing S, Tong H-W, Pei S-W, et al The earliest unequivocally modern humans in southern China. Nature. 2015; 526: 696–699. doi: 10.1038/nature15696 26466566 10.1038/nature15696

[81] Beven K. A manifesto for the equifinality thesis. Journal of Hydrology. 2006; 320: 18–36.

