# Supplementary material for: A serial founder effect model of phonemic diversity based on phonemic loss in low-density populations
Source: PLoS One. 2018 Jun 1;13(6):e0198346. doi: 10.1371/journal.pone.0198346 (PMC5983421; doi:10.1371/journal.pone.0198346)
Supplement: S1 Text — (DOCX) [file pone.0198346.s001.docx]

Pérez-Losada & Fort, A serial founder effect model of phonemic diversity based on phonemic loss in low-density populations.

**S1 Text. Supplementary results**

1. **The slope of the observed cline of *t_F_* does not depend on the distance interval**

In this section we explain how we have detected a cline of phonemic diversity *t_F_*. In Fig SA1, each bin gives the value of *t_F_* computed using all languages with distance within the corresponding interval. For example, for the upper left plot in Fig SA1, the first value of *t_F_* has been computed using all languages in the interval 0-1000 km, the second one using the languages in 1000-2000 km, etc. (this plot also appears in Fig 1 in the main paper). We see that using four different distance intervals in Fig SA1 (1000 km, 1500 km, 2000 km and 3000 km), we obtain essentially the same slope. This confirms that the value of the observed slope of the cline of phonemic diversity *t_F_* does not depend on the interval of distances chosen.


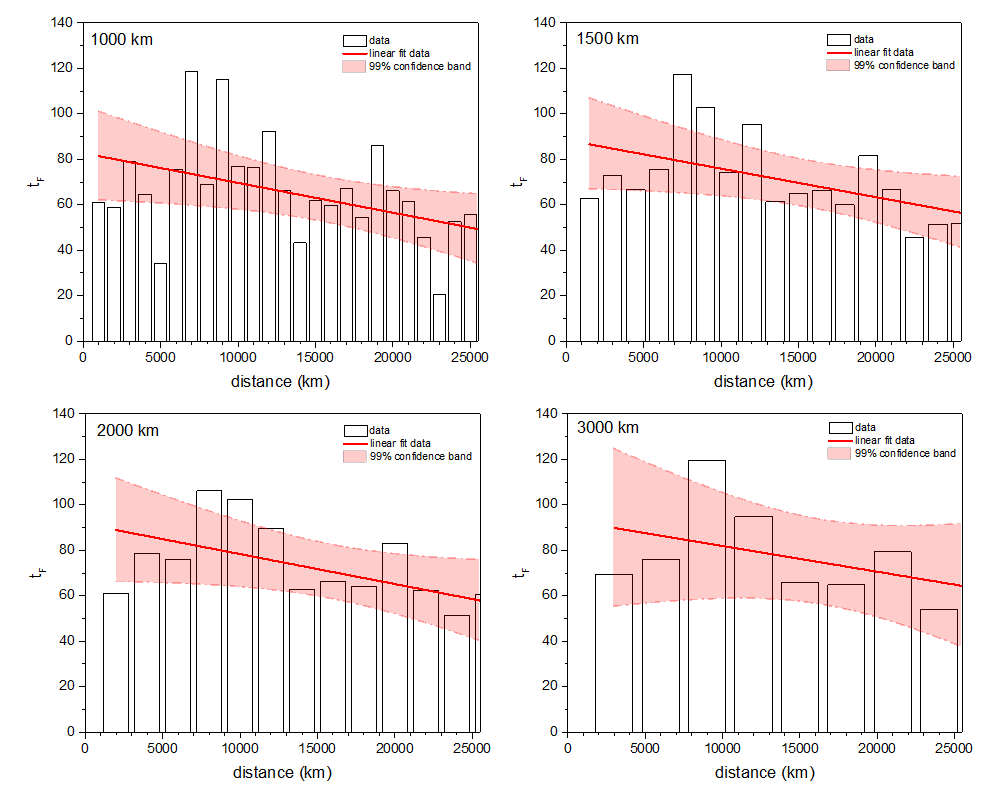


**Fig SA1. Observed diversity cline**. All of these plots have been obtained from the data (not the simulations). Plot of phonemic diversity, binned in 1000, 1500, 2000 and 3000 km intervals, versus the distance from origin, for the languages in our Database S1. The linear fit (red line) has slope= -(0.5-2.1)·10^-3^ km^-1^, *r* = -0.509 for 1000 km; slope= -(0.4-2.1)·10^-3^ km^-1^, *r* = -0.578, for 1500 km; slope= -(0.3-2.3)·10^-3^ km^-1^, *r* = -0.621 for 2000 km; and slope= -(0.4-2.7)·10^-3^ km^-1^, *r* = -0.525 for 3000 km. All ranges reported are 95% confidence-level intervals.

**B. Simulated cline for the number of phonemes corresponding to Fig 3a in the main paper**

In Figs 1-2 in the main paper we were interested in the number of phonemes. In Figs 1-2 we have used languages (i.e., lists of “1”s and “0”s) built by us, but the results would be the same if using languages from Database S1, because Figs 1-2 deal with the number of phonemes (not with *t_F_*). In contrast, Fig 3 in the main paper deals with the phonemic diversity *t_F_*. If there are several languages, in order to calculate *t_F_* we need the precise phonemes of each language (so that we can calculate the values of the relative frequencies *p_i_* appearing in Eq. (1) in the main paper). Therefore, as stated in the caption to Fig 3, in the simulations to measure *t_F_*, we begin with several languages drawn from Database S1.

In Fig 3a in the main paper, we show two simulated clines of diversity *t_F_* for *R_o_* = 1.2 (one for a phonemic loss time of 60 generations/phoneme, and another one for 120 generations/phoneme). They are consistent with the observed cline of diversity *t_F_*. Here we check that the same simulations also yield clines for the number of phonemes (instead of the diversity *t_F_*) that are consistent with the cline detected by Atkinson for the number of phonemes [1].

Figs SB1 and SB2 plot the number of phonemes vs distance for the same phonemic loss times as in Fig 3a, i.e. 60 (blue) and 120 (green) generations/phoneme, respectively. On the other hand, the range for the slope of the observed phonemic cline, namely -(3.4-6.5)·10^-4^ phonemes/km, is shown as the full, dashed-dotted and dotted red lines (95% confidence-level (CL) intervals from Fig 1 in Ref. [2]). The same simulation runs have been used to generate Figs 3a-b in the main paper. In both Figs SB1 and SB2, the simulated cline lies within the bounds of the observed cline. Although the magnitude of the slope in Fig. SB1 is a little larger than the observed range, clearly the simulated slope will be within the observed range for many values of the phonemic loss time between 80 and 120 generations/phoneme. We conclude that, for the simulations of the cline in Fig 3a, the number of phonemes also displays a cline which is consistent with that detected by Atkinson [1].

**Fig SB1. Number of phonemes versus distance for a short phonemic loss time.** Plot of the number of phonemes versus the distance from the origin of the out-of-Africa dispersal, for a phonemic loss time of 60 generations/phoneme. Net fecundity *R_0_* = 1.2. The linear fit (blue line) has slope= -7.65·10^-4^ phonemes/km, intercept = 39.7 phonemes, *r* = -0.978. At the start of the simulation only the central node of the grid is populated with a population of *N* = 10 tribes, speaking a mixture made of two initial languages (5 tribes speaking Ewe and 5 speaking Ngizim) with 40 phonemes each. The maximum number of tribes per node (*N_s_*) is set to 10. The demographic threshold below with phonemes are lost is also set to 10. Time = 2,280 generations, corresponding to the present time since the Out-of-Africa dispersal. The red lines are the cline detected by Atkinson [1] for present languages, and they have been plotted using the 95% CL ranges for the slope and intercept obtained in Ref. [2], Fig 1.

**Fig SB2. Number of phonemes versus distance for a long phonemic loss time.** Plot of the number of phonemes, versus the distance from the origin of the Out-of-Africa dispersal, for a net fecundity *R_0_* = 1.2. The linear fit (green line) has slope= -4.22·10^-4^ phonemes/km, intercept = 40.2 phonemes, *r* = -0.975. At the start of the simulation only the central node of the grid is populated with a population of *N* = 10 tribes, speaking a mix made of two initial languages (5 tribes speaking Ewe and 5 speaking Ngizim) with 40 phonemes each. The maximum number of tribes per node (*N_s_*) is set to 10. The demographic threshold below with phonemes are lost is also set to 10. Time = 2,280 generations, corresponding to the present time since the Out-of-Africa dispersal. The red lines are the cline detected by Atkinson [1] for present languages, and have been obtained using the 95% CL ranges for the slope and intercept in Ref. [2], Fig 1.

**C. Effect of the net fecundity *R_0_* on the simulated diversity cline**

Figs SC1 and SC2 show the simulated and observed phonemic diversities, measured as *t_F_* (binned on 1000 km intervals) versus distance, for two net fecundities (*R_o_* = 1.4 and *R_o_* = 1.3). Rectangles represent the observed phonemic diversity (measured as *t_F_*) of present languages (binned within 1000 km intervals) and the fitted regression is shown as a red line (as in Fig. 1 and SA1). At the start of the simulation, all cells are empty except the central one, which is populated with 10 tribes. Each tribe speaks one of two languages (5 tribes speak Ewe and 5 speak Ngizim) of 40 phonemes each. Our simulations (blue and green squares and regression lines) cannot reproduce the observed global diversity cline if *R_o_* is equal (or greater) than 1.4 (Fig SC1). Indeed, regardless the phonemic loss time tested, namely 60 (blue) and 120 (green) generations/phoneme, the simulated cline of *t_F_* in Fig SC1 yields a very small slope, namely -(0.26-0.43)·10^-3^ km^-1^ and -(0.05-0.13)·10^-3^ km^-1^ respectively (95% confidence-level intervals), whereas the observed range for the slope of the global phonemic cline of *t_F_* (red line) is -(0.5-2.1)·10^-3^ km^-1^. The reason is that saturation is reached faster for larger values of *R_o_*, thus less nodes are active (at a given time) when mutation (reduction of one randomly chosen phoneme of languages at cells with less tribes than the threshold value) is applied, resulting in a weaker reduction of phonemic diversity. Fig SC2 shows the clines obtained by reducing fecundity to *R_o_* = 1.3. A phonemic loss time of 60 generations/phoneme (blue line) yields a slope, namely -(1.0-1.2)·10^-3^ km^-1^ (95% confidence-level intervals), within the range of the observed cline -(0.5-2.1)·10^-3^ km^-1^ (red line). A larger phonemic loss time of 120 generations/phoneme (green line) yields -(0.41-0.49)·10^-3^ km^-1^ (95% confidence-level intervals), which does not overlap with the range of the observed slope. The reason is simply that the phonemic loss process is slower because the time to lose a phoneme (phonemic loss time) is larger, so the phonemic loss mechanism that generates the cline is weaker. A cline consistent with the observed one is still present if *R_o_* = 1.2 (Fig 3a in the main paper). If *R_o_* = 1.1, the cline of *t_F_* still appears (Fig SC3), but we have observed that the cline of the number of phonemes is substantially below the observed one (Fig SC4, left). Of course, this could be avoided by using initial languages with more phonemes, but understanding the reason will lead us to conclude that *R_0_* = 1.1 is not acceptable. The reason is that, for such a low net fecundity, some cells behind the population front are not saturated (Fig SC5, left) and, therefore, they continually loose phonemes. Obviously, a spatially oscillating population profile (Fig SC5, left) is not realistic, because we expect intuitively that colonized areas will be saturated (as in Fig SC5, right). In conclusion, the simulations lead to unacceptable results for values of *R_o_* larger than 1.4 and lower that 1.1. For 1.1 < *R_0_* < 1.4, we have checked that the cline of the number of phonemes is consistent with the observed one (Fig SC4, right, shows an example, obtained for *R_0_* = 1.3).

**Fig SC1. Diversity versus distance for *R_o_* = 1.4.** Plot of phonemic diversity, binned in 1000 km intervals, versus the distance from the origin of the Out-of-Africa dispersal, for a net fecundity of 1.4. The linear fit (red line) to the observed cline (histogram) has slope= -(0.5-2.1)·10^-3^ km^-1^, *r* = -0.509. The simulated slopes are -(0.26-0.43)·10^-3^ km^-1^, *r* = -0.859 (blue line, 60 generations/phoneme) and -(0.05-0.13)·10^-3^ km^-1^, *r* = -0.687 (green line, 120 generations/phoneme). At the start of the simulation, only the central node of the grid is populated with a population of *N* = 10 tribes, speaking a mix of 2 languages (5 tribes speaking Ewe and 5 speaking Ngizim) of 40 phonemes each. The maximum number of tribes per node (*N_s_*) is set to 10. The demographic threshold below with phonemes are lost is also set to 10. Time = 2,280 generations or about 70 kyr, corresponding to the present time since the Out-of-Africa dispersal.

**Fig SC2. Diversity versus distance for *R_o_* = 1.3.** Plot of phonemic diversity, binned in 1000 km intervals, versus the distance from the origin of the Out-of-Africa dispersal, for a net fecundity of 1.3. The simulations (blue and green dots) have been obtained using two initial languages (5 tribes speaking Ewe and 5 speaking Ngizim) with 40 phonemes each. The linear fit (red line) to the observed cline (histogram) has slope= -(0.5-2.1)·10^-3^ km^-1^, *r* = -0.509. The simulated slopes are -(1.0-1.2)·10^-3^ km^-1^, *r* = -0.977 (blue line, 60 generations/phoneme) and -(0.41-0.49)·10^-3^ km^-1^, *r* = -0.979 (green line, 120 generations/phoneme). At the start of the simulation, only the central node of the grid is populated, with a population of *N* = 10 tribes. The maximum number of tribes per node (*N_s_*) is set to 10. The demographic threshold below with phonemes are lost is also set to 10. Time = 2,280 generations or about 70 kyr, corresponding to the present time since the Out-of-Africa dispersal.

**Fig SC3. Diversity versus distance for *R_o_* = 1.1.** Plot of phonemic diversity, binned in 1000 km intervals, versus the distance from the origin of the Out-of-Africa dispersal, for a net fecundity of 1.1. Two initial languages (5 tribes speaking Ewe and 5 speaking Ngizim) with 40 phonemes each. The linear fit (red line) to the observed cline (histogram) has slope= -(0.5-2.1)·10^-3^ km^-1^, *r* = -0.509. The linear fit to the simulations (blue line) for 60 generations/phoneme has slope -(0.9-1.0)·10^-3^ km^-1^, *r* = -0.984. At the start of the simulation only the central node of the grid is populated, with a population of *N* = 10 tribes. The maximum number of tribes per node (*N_s_*) is set to 10. The demographic threshold below with phonemes are lost is also set to 10. Time = 2,280 generations or about 70 kyr, corresponding to the present time since the Out-of-Africa dispersal.


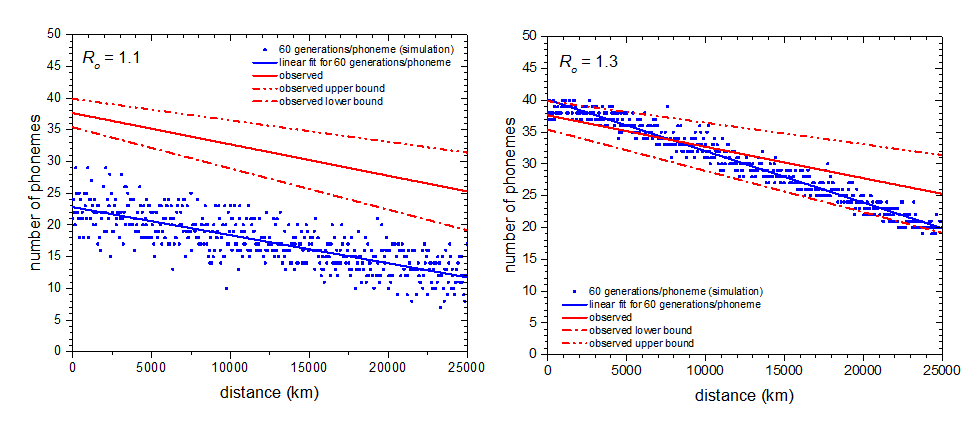


**Fig SC4. Number of phonemes versus distance for *R_o_* = 1.1 and *R_o_* = 1.3.** Plot of the number of phonemes, versus the distance from the origin of the Out-of-Africa dispersal, for a phonemic loss time of 60 generations/phoneme. The simulations (blue dots) in both figures have been obtained using two initial languages (5 tribes speaking Ewe and 5 speaking Ngizim) with 40 phonemes each. At the start of the simulation only the central node of the grid is populated with a population of *N* = 10 tribes. The maximum number of tribes per node (*N_s_*) is set to 10. The demographic threshold below with phonemes are lost is also set to 10. Time = 2,280 generations or about 70 kyr, corresponding to the present time since the Out-of-Africa dispersal.


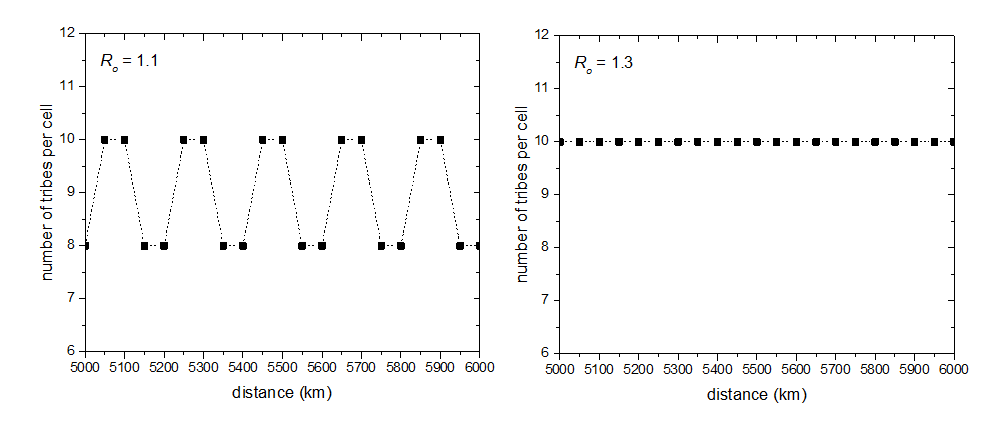


**Fig SC5. Number of tribes per cell versus distance for *R_o_* = 1.1 and *R_o_* = 1.3.** Plot of the number of tribes per cell, versus the distance from the origin of the Out-of-Africa dispersal, at time = 2280 generations and a phonemic loss time of 60 generations/phoneme. Note that some nodes are below saturation if R_o_ = 1.1 (left) while all nodes are saturated for R_0_ = 1.3 (right). At the start of the simulation only the central node of the grid is populated with a population of *N* = 10 tribes. The maximum number of tribes per node (*N_s_*) is set to 10. The demographic threshold below with phonemes are lost is also set to 10. Time = 2,280 generations or about 70 kyr, corresponding to the present time since the Out-of-Africa dispersal. These simulation runs are the same as these that have been used to obtain Fig SC4.

**D. Effect of the number of initial languages on the simulated diversity cline**

In the previous section, we have determined the range of net fecundity values *R_0_* that produce phonemic clines consistent with the observed one. Now we want to elucidate whether the diversity cline arises regardless of the number of languages present at the start of the simulation or not. This is a relevant topic, because it may help to introduce a quantitative framework on the debate of the initial number of languages at the onset of the Out-of-Africa dispersal [3-5]. Figs SD1 and SD2 show the phonemic diversity (measured as *t_F_*) versus distance for two examples of 1 single initial language, with 40 and 77 phonemes respectively. The phonemic diversity cline is present for the two phonemic loss times tested: a shorter phonemic loss time of 60 generations/phoneme (blue line), and a longer time of 120 generations/phoneme (green line). In the second case, the time needed to lose a phoneme is larger, so the effect (slope of the cline) is weaker. We note that for a single initial language of 40 phonemes (Fig SD1), the intercepts of the simulations (blue and green lines) are smaller than the intercept of the observed cline (red line). However, the simulated and observed intercepts agree for, e.g., a single initial language of 77 phonemes (Fig SD2). The reason is simply that the higher the number of phonemes, the higher its diversity, i.e. the value of the intercept of the simulated cline of *t_F_*. Thus, the important issue is the slope and not the intercept, because the intercept depends only on the initial language(s), whereas the slope depends on the mechanisms driving the dynamics of the system (dispersal distance, phonemic loss time, reproduction rate, etc.).

For a single initial language, the slope of the simulated cline is clearly smaller than the observed slope if the phonemic loss time is large (120 generations/phoneme, green squares and line in Figs SD1 and SD2). However, the slope of the simulated cline is consistent with the observed one if the phonemic loss time is small (60 generations/phoneme, blue in Figs SD1 and SD2). Fig 3a in the main paper shows the diversity cline for an example of two initial languages with 40 phonemes each. In this case, again, the simulated slope for a phonemic time loss of 60 generations/phoneme (blue) is consistent with the observed one (red line). Fig SD3 shows the case of 10 initial languages. Again, a cline consistent with the observed one is obtained for a phonemic loss time of 60 generations/phoneme (blue squares and line).

We conclude that, irrespective of the number of initial languages, the model can yield clines of diversity *t_F_* consistent with the data.

**Fig SD1. A single initial language of 40 phonemes.** Plot of phonemic diversity, binned in 1000 km intervals, versus the distance from the origin of the Out-of-Africa dispersal, for a net fecundity of 1.2. The linear fit (red line) to the observed cline has slope= -(0.5-2.1)·10^-3^ km^-1^, *r* = -0.509. The simulated slopes are -(0.68-0.78)·10^-3^ km^-1^, *r* = -0.986 (blue line, 60 generations/phoneme) and -(0.32-0.38)·10^-3^ km^-1^, *r* = -0.980 (green line, 120 generations/phoneme). At the start of the simulation only the central node of the grid is populated with a population of *N* = 10 tribes, speaking a single language of 40 phonemes (Ewe). The maximum number of tribes per node (*N_s_*) is set to 10. The demographic threshold below with phonemes are lost is also set to 10. Time = 2,280 generations or about 70 kyr, corresponding to the present time since the Out-of-Africa dispersal.

**Fig SD2. A single initial language of 77 phonemes.** Plot of phonemic diversity, binned in 1000 km intervals, versus the distance from the origin of the Out-of-Africa dispersal, for a net fecundity of 1.2. The linear fit (red line) to the observed cline has slope= -(0.5-2.1)·10^-3^ km^-1^, *r* = -0.509. The simulated slopes are -(0.66-0.80)·10^-3^ km^-1^, *r* = -0.974 (blue line, 60 generations/phoneme) and -(0.26-0.31)·10^-3^ km^-1^, *r* = -0.982 (green line, 120 generations/phoneme). At the start of the simulation only the central node of the grid is populated with a population of *N* = 10 tribes, speaking a single language of 77 phonemes (Puk). The maximum number of tribes per node (*N_s_*) is set to 10. The demographic threshold below with phonemes are lost is also set to 10. Time = 2,280 generations or about 70 kyr, corresponding to the present time since the Out-of-Africa dispersal.

**Fig SD3. Ten initial languages.** Plot of phonemic diversity, binned in 1000 km intervals, versus the distance from the origin of the Out-of-Africa dispersal, for a net fecundity of *R_0_* = 1.2. The linear fit (red line) to the observed cline has slope= -(0.5-2.1)·10^-3^ km^-1^, *r* = -0.509. The simulated slopes are -(1.7-1.8)·10^-3^ km^-1^, *r* = 0.994 (blue line, 60 generations/phoneme) and -(0.28-0.41)·10^-3^ km^-1^, *r* = 0.839 (green line, 120 generations/phoneme). At the start of the simulation only the central node of the grid is populated with a population of *N* = 10 tribes, speaking a randomly generated mix of ten languages within from Database S1 within a range of 35 to 40 phonemes. The maximum number of tribes per node (*N_s_*) is set to 10. The demographic threshold below with phonemes are lost is also set to 10. Time = 2,280 generations or about 70 kyr, corresponding to the present time since the Out-of-Africa dispersal.

**E. Effect of time elapsed from the onset of the out-of-Africa dispersal**

It is of interest to know how the clines of diversity *t_F_* and number of phonemes evolve in time, for several reasons. First, there is a current debate on when and how many Out-of-Africa dispersals occurred [6,7]. Second, some authors have hypothesized that a phonemic signal attributable to a serial founder effect (SFE) from the out of Africa dispersal would perhaps have disappeared long ago [8-10]. In order to see how the signal evolves in our model, we performed simulations with large values of time. Fig SE1 shows the phonemic diversity (measured as *t_F_*) versus distance at several times. Time t = 1168 generations (black squares) is when the population front reaches the border of the square grid (at 25000 km). From this time on, as the entire grid of cells is saturated, the phonemic loss mechanism ceases to create diversity. All other simulations in this Text S1 (and all simulations in the main paper) have been performed up to time = 2280 generations, or 72960 years (red squares in Figs SE1-2), which would correspond to the present time. After the population reaches the edges of the simulation grid (at t = 1168 generations), there is no phonemic loss neither net reproduction, so the only process taking place is the movement of randomly chosen tribes (languages) between neighboring cells. This process tends to homogenize the system, and therefore to gradually erase both the cline of diversity *t_F_* (Figs SE1-2) and the cline of the number of phonemes (Fig SE3). However, we can see that the signal is still very clear at a time of t = 9120 generations or 300 kyr (olive squares) i.e., much later than today. Fig SE2 shows the phonemic diversity (measured as *t_F_*), computed in 1000 km intervals, versus distance. The presence of the signal is more clearly seen. Fig SE3 plots the fitted straight lines to the number of phonemes per node (not to diversity *t_F_*) versus distance for the same times. We can consistently see the presence of a phonemic cline, which becomes erased much more slowly than the diversity cline (Figs SE1-2). The important point is that, even for much longer times since the out-of-Africa dispersal than the present one, both clines still persist.

**Fig SE1. The cline of phonemic diversity *t_F_* is gradually erased in time (arrow).** Plot of phonemic diversity versus the distance from the origin of the Out-of-Africa dispersal, at several simulation times, for a net fecundity of *R_0_* = 1.2. In this figure, each value of *t_F_* has been computed for the 10 tribes in the corresponding cell. At the start of the simulation only the central node of the grid is populated with a population of *N* = 10 tribes speaking a single language of 35 phonemes. The maximum number of tribes per node (*N_s_*) is set to 10. The demographic threshold below with phonemes are lost is also set to 10.

**Fig SE2. The cline of phonemic diversity *t_F_* is gradually erased in time (arrow).** Plot of phonemic diversity versus the distance from the origin of the Out-of-Africa dispersal, at several simulation times, for a net fecundity of *R_0_* = 1.2. In this figure, each value of *t_F_* has been computed for all simulated languages within a ring of width 1000 km centered at the origin of the dispersal (as for the squares in Figs 3a, SC1-3 and SD1-3). At the start of the simulation only the central node of the grid is populated with a population of *N* = 10 tribes speaking a single language of 35 phonemes. The maximum number of tribes per node (*N_s_*) is set to 10. The demographic threshold below with phonemes are lost is also set to 10.

**Fig SE3. The cline of the number of phonemes is gradually erased in time (arrow).** Plot of linear fit of the total number of phonemes versus the distance from the origin of the Out-of-Africa dispersal, at several simulation times, for a net fecundity of *R_0_* = 1.2. The black line cannot be seen because it is behind the red one. At the start of the simulation only the central node of the grid is populated with a population of *N* = 10 tribes speaking a single language of 35 phonemes. The maximum number of tribes per node (*N_s_*) is set to 10. The demographic threshold below with phonemes are lost is also set to 10.

**F. Effect of the demographic threshold of phonemic loss**

The demographic threshold parameter (*DT*) gives the number below which the cells will be active, meaning that languages on these cells will lose one phoneme each time increment equal to the phonemic loss time. For instance, if *DT* = 3, only cells with less than 3 tribes will be active (i.e., will lose phonemes). For all simulations reported in the main paper, the threshold is set to *DT* = 10. Recall that the maximum number of tribes per square cell is also set to *N_s_* = 10. Therefore, in the main paper all cells except the saturated ones loose phonemes. Then, we know from Fig 2 in the main paper that phonemic loss times above 50 generations/phoneme yield clines with slope consistent with the observed one (horizontal, dashed and dashed-dotted lines in Fig 2), and that the best agreement with the simulations is reached for a phonemic loss time of about 80 generations/phoneme (Fig 2 in the main paper). This is also seen in Fig SF1 for (*DT* = 10 tribes). We have repeated the simulations for lower values of the *DT* threshold (Fig SF1). We observe that the lower the threshold, the faster the necessary rate (i.e., less generations per lost phoneme) to obtain the slope of the observed cline. This is reasonable because if the threshold is lower, a smaller percentage of tribes lose phonemes, so a faster rate is necessary to lose the same number of phonemes.

Fig SF1. Plot of phonemic loss times which gives the best agreement to the observed slope (for the number of phonemes versus distance from the origin of the Out-of-Africa dispersal), versus the demographic threshold, for a net fecundity of *R_0_* = 1.4. At the start of the simulation only the central node of the grid is populated with a population of *N* = 10 tribes, speaking a randomly generated mix of synthetically generated languages within a range of 35 to 40 phonemes. The maximum number of tribes per node (*N_s_*) is set to 10. The red line is the linear fit.

**G. Effect of the saturation number of tribes per node**

In the main paper we have used ethnographically observed values of the population density and number of individuals per tribe to estimate *N_s_* = 10 tribes per node of 50 km x 50 km. However, some hunter-gatherers have substantially higher population densities [2]. Therefore, in Figs SG1-2 we also consider the cases of *N_s_* = 15 and *N_s_* = 20 tribes per node. We see that the maximum density population does not affect the overall trend of the simulated slopes of the number of phonemes (Fig SG1) and the phonemic diversity *t_F_* (Fig SG2). For all cases, the phonemic loss time that agrees better with the observed cline of the number of phonemes (horizontal lines in Fig SG1) is between 70 and 120 generation/phonemes (similarly to Fig 2 in the main paper). Thus, we can conclude that the slope of the simulated cline of the number of phonemes is quite insensitive to the maximum population density. In other words, the simulated cline of the number of phonemes does not depend on the number of tribes per node assumed. For the phonemic diversity (Fig SG2), the slope from the simulations that agrees better with the observed cline of the phonemic diversity (horizontal lines) is always between 50 and 120 generations/phoneme. Thus in our main paper the conclusions are independent of the selected number of tribes per node.

**Fig SG1. Number of phonemes versus distance.** Slope for the cline of the number of phonemes, obtained from simulations varying the maximum (saturation) number of tribes per node, as a function of the phonemic loss time, for a net fecundity of *R_0_* = 1.2. Horizontal lines are the mean observed slope, and its lower and upper bounds from Fig 1 in Ref. [1]. For all simulations reported, the demographic threshold is set to 10 (for other values, see the previous section). All simulations were populated with an initial set of 10 languages of 30-40 phonemes.

**Fig SG2. Diversity *t_F_* versus distance**. Slope for the cline of phonemic diversity *t_F_*, obtained from simulations varying the maximum (saturation) number of tribes per node, as a function of the phonemic loss time, for a net fecundity of *R_0_* = 1.2. Horizontal lines are the mean observed slope, and its lower and upper bounds from Fig SA1. For all simulations reported, the demographic threshold is set to 10 (for other values, see the previous section). All simulations were populated with an initial set of 10 languages of 30-40 phonemes.

**H. Simulation model with no phonemic loss**

Founder events are defined as the loss of cultural diversity (phonemes in our case) due to random sampling in small populations. However, in our simulations of the global phonemic cline, there is an additional effect, namely a phonemic loss rate in low-diversity populations. Therefore, the question arises if a cline can be generated by founder effects alone, i.e. due to sampling effects when low-density populations reproduce (at the leading, low-density edge of the advancing front). In order to answer this question, here we show some results in the absence of phonemic loss (this would correspond to the limit of an infinite phonemic loss time). In this way, the effect of fission (serial founder events) can be analyzed without the simultaneous effect of mutation (phonemic loss). Therefore, in this section languages do not evolve. Initially (i.e., at the onset of the out‐of‐Africa dispersal) there are 10 tribes speaking 5 different languages in the central cell of the grid (with 35, 36, 37, 38 and 40 phonemes). In Fig SH1 we observe that without phonemic loss, the simulations do not generate any geographic phonemic cline of the type reported by Atkinson [1]. Thus not only fission (drift or founder effects), but also a phonemic loss rate is needed to obtain a cline similar to the observed one. Without phonemic loss, the number of phonemes of each language remains constant. But drift or fission (serial founder effect) is still present. This cannot decrease the number of phonemes in each language, but it can certainly lead to a loss of languages and, therefore, to a reduction of the number of phonemes per unit area. However, Atkinson detected a reduction of the number of phonemes per language as a function of distance. This loss of languages can indeed be seen in the snapshots in Fig SH1. In the first figure (t = 500 generations), the front has propagated up to about 10,000 km. We can observe that near the origin (left-hand side), which corresponds to the center of the simulation grid, all 5 initial languages are present. As we move along the horizontal axis, away from the center, a different picture appears. At distances of about 5,000 km, only two languages are present (with 38 and 35 phonemes). This also happens at later times (Fig SH1), so fission (founder effects) reduces the number of languages per unit area. Note that a language that does not appear at the right-hand side of Fig SH1 for t = 2280 generations, has not necessarily disappeared. It can be present in the grid, not only at small distances, but possibly also at large distances in other directions different from the positive horizontal axis.

Whereas Atkinson detected a global cline of decreasing number of phonemes per language, the simulations without phonemic loss (Fig SH1) do not predict such a cline, although they do predict that some languages may not propagate at large distances. In conclusion, spatial drift per se cannot explain the observed global cline.


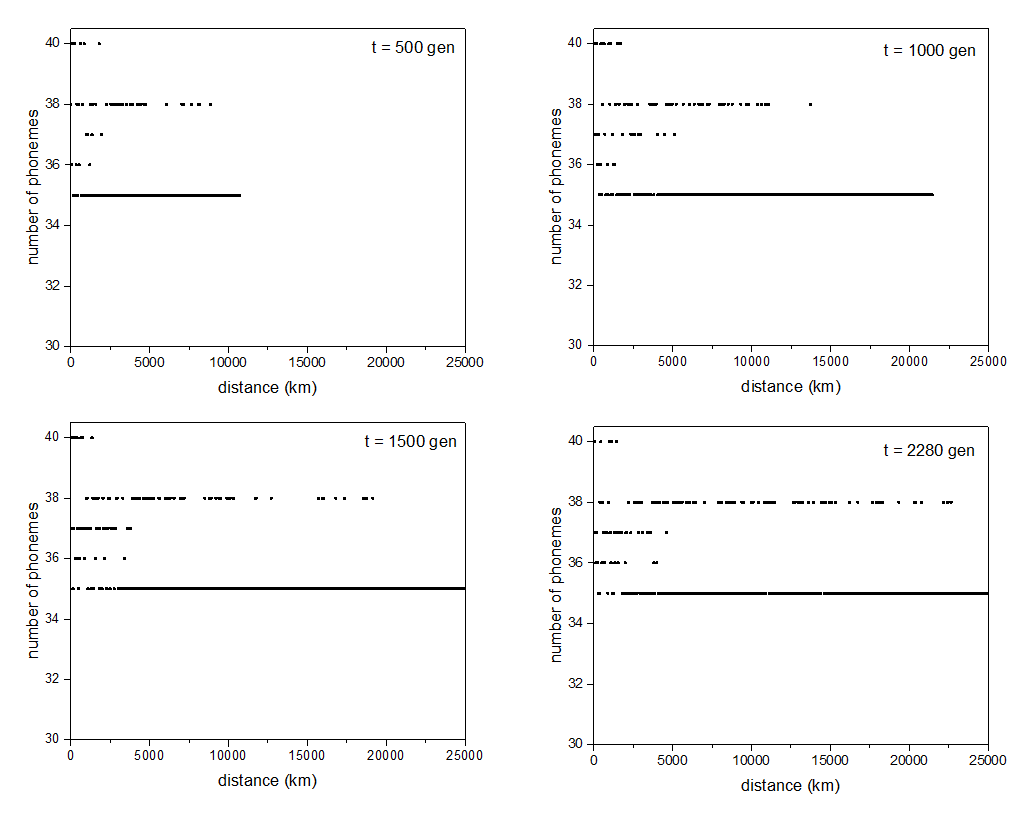


Fig SH1. An example of results from a single simulation run of our model without phonemic loss, along the horizontal positive axis. Note that at large distances from the origin, a single language is selected.

**I. Effect of fast migrations on the simulated global phonemic diversity cline**

The slopes of the simulations give information on the intensity of phonemic change over distance. In our simulations, as in previous work [2], in order to attain reasonable computing times, we considered that the dispersal distance per generation is either 0 km or d = 50 km. The value d = 50 km is representative of pre-industrial populations [11]. In this section, we want to know the effect of longer dispersal distances [12] on both the phonemic cline and the phonemic diversity cline. Obviously, longer dispersal distances per generation will lead to faster fronts. Therefore, in Figs SI1 and SI2 we consider the cases of *d* = 100 km (left) and *d* = 200 km (right). Fig SI1 shows that, for the range tested, the dispersal distance does affect the overall trend of the slope of the phonemic diversity cline. For all cases, regardless the phonemic loss time, the model predicts a virtually horizontal slope of the phonemic cline. The effect is more pronounced for larger distances (right). We conclude that such large values of the dispersal per generation (d = 100 km or d = 200 km) are inconsistent with the observed cline of diversity *t_F_*. On the other hand, in Fig SI2 the slopes of the clines for the number of phonemes (not the diversity *t_F_*) versus distance lie within the slope range of the observed phonemic cline. Thus, we can conclude that fast migrations (due to large dispersal distances) can lead to a cline for the number of phonemes consistent with that observed by Atkinson, but not to a cline for the phonemic diversity *t_F_* consistent with that observed by us.


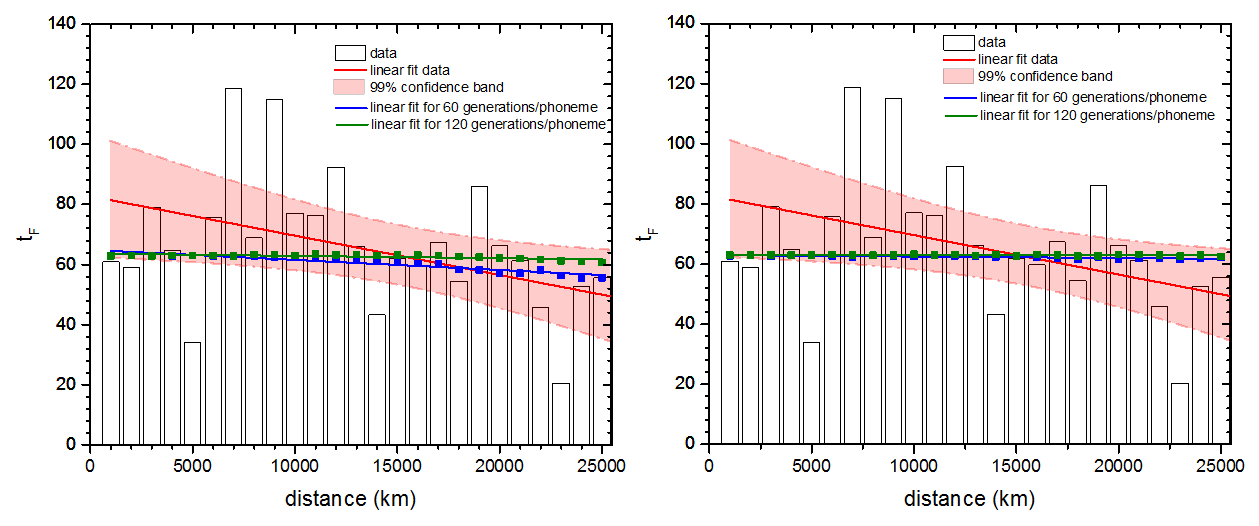


**Fig SI1. Diversity *t_F_* versus distance for dispersal distances *d* = 100 km (left) and *d* = 200 km (right).** Plot of phonemic diversity *t_F_*, binned in 1000 km intervals, versus the distance from the origin of the Out-of-Africa dispersal, for a net fecundity of 1.2. The simulations (blue and green dots) in both figures have been obtained using two initial languages (Ewe and Ngizim) with 40 phonemes each, and a phonemic loss time of 60 generations/phoneme (blue) or 120 generations/phoneme (green). The linear fit (red line) to the observed cline has slope= -(0.5-2.1)·10^-3^ km^-1^, *r* = -0.509. For *d* = 100 km (left), the simulated slopes are -(2.8-3.9)·10^-4^ km^-1^, *r* = -0.936 (blue line, 60 generations/phoneme) and -(0.4-1.1)·10^-4^ km^-1^, *r* = -0.728 (green line, 120 generations/phoneme). For *d* = 200 km (right), the simulated slopes are -(3.4-6.6)·10^-4^ km^-1^, *r* = -0.791 (blue line, 60 generations/phoneme) and -(0.2-1.9)·10^-5^ km^-1^, *r* = -0.327 (green line, 120 generations/phoneme). Note that all of these simulated slopes are too small compared to the observed range. At the start of the simulation only the central node of the grid is populated with a population of *N* = 10 tribes. The maximum number of tribes per node (*N_s_*) is set to 10. The demographic threshold below with phonemes are lost is also set to 10. Time = 2,280 generations or about 70 kyr, corresponding to the present time since the Out-of-Africa dispersal.


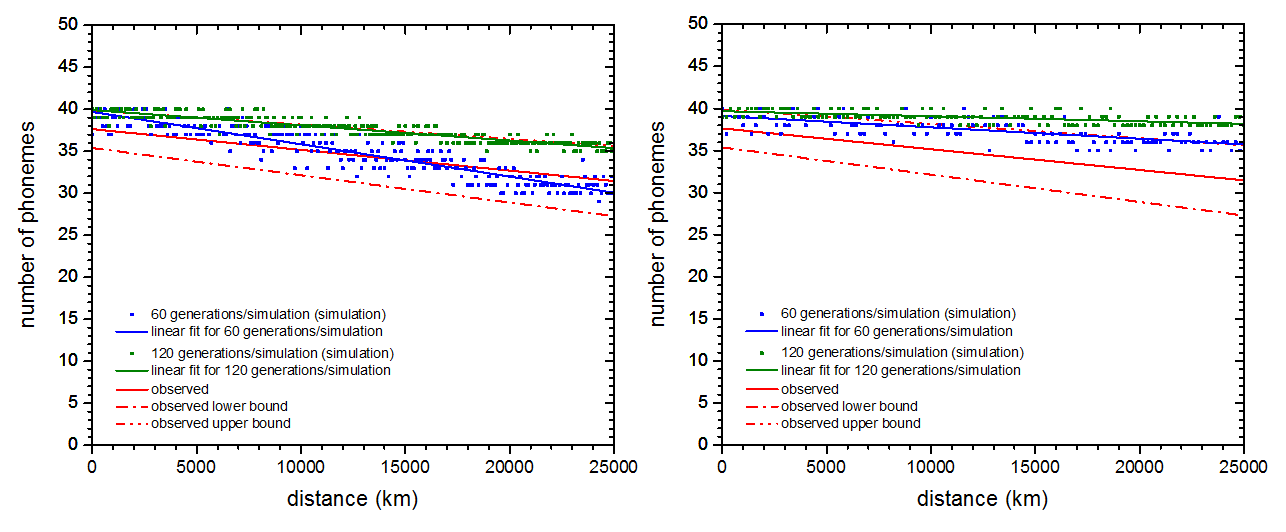


**Fig SI2. Number of phonemes versus distance for dispersal distances *d* = 100 km (left) and *d* = 200 km (right).** Plot of the number of phonemes, versus the distance from the origin of the Out-of-Africa dispersal, for a net fecundity of 1.2. The simulations (blue and green dots) in both figures have been obtained using two initial languages (Ewe and Ngizim) with 40 phonemes each. At the start of the simulation only the central node of the grid is populated with a population of *N* = 10 tribes. The maximum number of tribes per node (*N_s_*) and the demographic threshold are set to 10. The demographic threshold below with phonemes are lost is also set to 10. Time = 2,280 generations or about 70 kyr, corresponding to the present time since the Out-of-Africa dispersal.

**J. The distribution of simulated slopes of the number of phonemes versus distance is Gaussian**

In the main paper, each symbols and error bar in Fig 2 gives a mean and twice a standard deviation σ, computed from the slopes obtained from 100 simulation runs (using the same parameter values). In the Results and Discussion, we state that a ±2σ error corresponds to a 95% confidence-level interval. This assumes that the distribution of the slopes is Gaussian. Here we find the distribution of slopes and ask if the Gaussian assumption is justified. Fig SJ1 has been obtained from the same 100 runs used to obtain the error bar in Fig 2 for initial languages of 35-40 phonemes (black square and error bar) and a phonemic loss time of 80 generations/phoneme (an example of such simulations also appears in Fig 1a). For all normality tests performed (Shapiro-Wilk, Lilliefors, Kolmogorov-Smirnov and Kolmogorov-Smirnov), at the 95% level, the data was significantly drawn from a normally distributed population. This implies that, within the 95% confidence level, our error bars of ±2σ (Fig 2) are justified.

**Fig SJ1. Frequency count versus slope for Fig 2 of main paper.** Plot of the number of counts, versus the slope of the linear fit to the simulated number of phonemes versus distance from the most likely origin of the Out-of-Africa dispersal, for initial languages of 35-40 phonemes and a phonemic loss time of 80 generations/phoneme. The distribution of slopes is normal.

**References**

1. Atkinson, QD. Phonemic diversity supports a serial founder effect model of language expansion from Africa. Science. 2011; 332, 346–349.
2. Fort J, Pérez-Losada J. Can a linguistic serial founder effect originating in Africa explain the worldwide phonemic cline? J R Soc Interface. 2016; 13: 20160185, 1-9.
3. Ruhlen M. The origin of language: tracing the evolution of the mother tongue. 1st ed. Wiley; 1994.
4. Coupé C, Hombert, J-M. Polygenesis of linguistic strategies: a scenario for the emergence of language. In: Minett J, Shi-Yuan Wang W, editors. Language Acquisition, Change and Emergence: essays in evolutionary linguistics, Chapter: 3. Hong Kong: City University of Hong Kong Press; 2005. pp. 153-201.
5. Nichols J. Monogenesis or polygenesis: a single ancestral language for all humanity? In: Gibson KR, Tallerman M, editors. The Oxford handbook of language evolution. Oxford, UK: Oxford University Press; 2013. pp. 559-572.
6. Reyes-Centeno H, Hubbe M, Hanihara T, Stringer C, Harvati K. Testing modern human Out-of-Africa dispersal models and implications for modern human origins. Journal of human evolution. 2015; 87(10): 1016.
7. Reyes-Centeno H, Rathmann H, Hanihara T, Harvati K. Testing modern human Out-of-Africa dispersal models using dental nonmetric data. Current Anthropology. 2017; 58(S17): S406-S417.
8. Gray RD. Evolution. Pushing the time barrier in the quest for language roots. Science. 2005; 309: 2007–2008.
9. Comrie B. Is there a single time depth cut-off point in historical linguistics? In: Renfrew C, McMahon A, Trask L, editors. Time depth in historical linguistics Vol 1. Cambridge, UK: The McDonald Institute for Archeological Research; 2000. pp. 33–43.
10. Reyes-Centeno H, Harvati K, Jäger G. Tracking modern human population history from linguistic and cranial phenotype. Scientific Reports. 2016; 6: 36645.
11. Fort J, Pérez-Losada J, Isern N. Fronts from integrodifference equations and persistence effects on the Neolithic transition. Phys Rev E. 2007; 76(3): 031913.
12. MacDonald DH. Hunter-gatherer mating distance and early-paleoindian social mobility. Current Research in the Pleistocene. 1997; 14: 119-121.
